# Blockade of phosphotyrosine pathways suggesting SH2 superbinder as a novel therapy for pulmonary fibrosis

**DOI:** 10.7150/thno.72269

**Published:** 2022-05-26

**Authors:** Meng Wang, An-Dong Liu, Qian Niu, Xiao Feng, Yuan-Yi Zheng, Shuai-Jun Chen, Hui Xu, Qian Li, Guo-Qing Hou, Xiao-Yang Bi, Yu-Zhi Lu, Pei-Pei Cheng, Li-Mei Liang, Ye-Han Jiang, Li-Qin Zhao, Fei Liu, Lin-Jie Song, Li-Ling Zhou, Ling-Yan Xiao, Feng Chen, Shawn Shun-Cheng Li, Wan-Li Ma, Xuan Cao, Hong Ye

**Affiliations:** 1 Department of Pathophysiology, School of Basic Medicine, Tongji Medical College, Huazhong University of Science and Technology, Wuhan, China; 2 Department of Medical Genetics, School of Basic Medicine, Tongji Medical College, Huazhong University of Science and Technology, Wuhan, China; 3 Department of Respiratory and Critical Care Medicine, Union Hospital, Tongji Medical College, Huazhong University of Science and Technology, Wuhan, China; 4 Department of Pathology, School of Basic Medicine, Tongji Medical College, Huazhong University of Science and Technology, Wuhan, China; 5 Department of Oncology, Tongji Hospital, Tongji Medical College, Huazhong University of Science and Technology, Wuhan, China; 6 Department of Forensic Medicine, School of Basic Medicine, Nanjing Medical University, Nanjing, China; 7 Department of Biochemistry, Schulich School of Medicine and Dentistry, Western University, London, Ontario, Canada; 8 Key Laboratory of Respiratory Diseases, National Health Commission of China, Wuhan, China

**Keywords:** IPF, phosphotyrosine (pY), fibroblast, SH2 superbinder, therapy

## Abstract

**Background:** Idiopathic pulmonary fibrosis (IPF) is a progressive and irreversible fibrotic disease with high mortality. Currently, pirfenidone and nintedanib are the only approved drugs for IPF by the U.S. Food and Drug Administration (FDA), but their efficacy is limited. The activation of multiple phosphotyrosine (pY) mediated signaling pathways underlying the pathological mechanism of IPF has been explored. A Src homology-2 (SH2) superbinder, which contains mutations of three amino acids (AAs) of natural SH2 domain has been shown to be able to block phosphotyrosine (pY) pathway. Therefore, we aimed to introduce SH2 superbinder into the treatment of IPF.

**Methods:** We analyzed the database of IPF patients and examined pY levels in lung tissues from IPF patients. In primary lung fibroblasts obtained from IPF patient as well as bleomycin (BLM) treated mice, the cell proliferation, migration and differentiation associated with pY were investigated and the anti-fibrotic effect of SH2 superbinder was also tested. *In vivo*, we further verified the safety and effectiveness of SH2 superbinder in multiple BLM mice models. We also compared the anti-fibrotic effect and side-effect of SH2 superbinder and nintedanib *in vivo*.

**Results:** The data showed that the cytokines and growth factors pathways which directly correlated to pY levels were significantly enriched in IPF. High pY levels were found to induce abnormal proliferation, migration and differentiation of lung fibroblasts. SH2 superbinder blocked pY-mediated signaling pathways and suppress pulmonary fibrosis by targeting high pY levels in fibroblasts. SH2 superbinder had better therapeutic effect and less side-effect compare to nintedanib *in vivo*.

**Conclusions:** SH2 superbinder had significant anti-fibrotic effects both *in vitro* and in *vivo*, which could be used as a promising therapy for IPF.

## Introduction

Pulmonary fibrosis is a chronic and irreversible lung interstitial disease caused by various pathogenic factors, including genetics, smoking and environmental exposures. Idiopathic pulmonary fibrosis (IPF), as the most complex type of pulmonary fibrosis, displays disrupted normal lung structure and the patients have very low five-year survival rate after diagnosis 1-3. The lung tissues of IPF patients have excess deposition of extracellular matrix (ECM), which may cause severe respiratory dysfunctions. Although the underlying etiology remains unclear, IPF was shown to be a result from the injury of alveolar epithelial type II cells, abnormal proliferation, migration, and differentiation of lung fibroblasts 4-7. Currently, there are only two valid small-molecule medications, nintedanib and pirfenidone, approved by the U.S. Food and Drug Administration (FDA), however they only have limited efficacy in IPF treatment 2. Thus, it is of high priority to understand the mechanisms of IPF and subsequently develop novel and effective medications.

Transforming growth factor-β (TGF-β) is well established signaling pathway, which is proved to be associated with fibrosis. However, drugs directly targeting TGF-β pathway (e.g. pirfenidone) have weak therapeutic effects 8, 9. Additionally, more potential factors contribute to IPF, such as cytokines, growth factors and hormones, which is likely to induce fibrosis mainly via binding to receptors evoked phosphorylation signaling especially phosphotyrosine (pY) 10. Tyrosine kinase (TK), which is composed of receptor tyrosine kinases (RTKs) and non-receptor tyrosine kinases (NRTKs), specifically phosphorylates tyrosines. Once ligands binding, intracellular tyrosine residues of RTKs are autophosphorylated, which is followed by recruitment of downstream signaling proteins containing Src homology-2 (SH2) or phosphotyrosine-binding (PTB) domains. This process leads to the activation and translocation of transcript factors to nuclei 11, 12. Binding of SH2 domain to pY residues determines the pattern of TKs-mediated signal transduction. Proteins containing SH2 domain play a central role in pY-based signal transduction 13. Therefore, we hypothesized that disrupting the association of pY and SH2 domain would block TKs-mediated signaling pathways and further inhibit fibrotic signal cascades.

SH2 superbinder (SH2 TrM) was first created by Kaneko and colleagues 14. It contains triple amino acids (AAs) mutants compared to wild-type SH2 domain of proto-oncogene tyrosine-protein kinase Src (SRC) (SH2 WT), and therefore has a stronger affinity to pY than SH2 WT 14. Moreover, SH2 TrM binds to pY regardless of AA sequences 15, 16, which results in blocking multiple TK-mediated signaling pathways. As TKs-mediated signaling pathways play an essential role in IPF 17-19, we hypothesized that SH2 superbinder could be a promising treatment for IPF. However, SH2 superbinder alone hardly crosses the cell membranes and accesses intracellular targets because of its hydrophilic properties. We modified SH2 superbinder with Nona-arginine (Arg)9, a cell penetrating peptide (CPP), to allow it pass through cell membranes effectively 20. Our previous research had proved that this modification could facilitate the penetration of SH2 superbinder into mouse melanoma cells (B16F10) *in vitro* and significantly suppressed tumor progression through blocking pY mediated signaling pathways 21. In this study, we hope to introduce GST-SH2 TrM-(Arg)9 to the treatment of pulmonary fibrosis.

## Methods

### Human subjects

Our study protocol was approved by the Institutional Review Board of the Union Hospital, Tongji Medical College, Huazhong University of Science and Technology. Informed consent was obtained from all subjects. The subjects include 3 patients with IPF and 3 subjects as control (normal lung tissues resected from adenocarcinoma). Patient information was shown in Table S1.

### Antibodies and reagents

Antibodies against FN (Cat#15613-1-AP), COL1A1 (Cat#67288-1-lg), α-SMA (Cat#55135-1-AP, Cat#67735-1-AP), FGFR1 (Cat#60325-1-lg), CD31 (Cat#60287-1-lg), CD45 (Cat#11265-1-lg), EpCAM (Cat#60287-1-lg), Vimentin (Cat#10366-1-AP) and GAPDH (Cat#60004-1-lg) were purchased from Proteintech (Rosemont, IL, USA). Antibodies against p-EGFR (Cat#3777S), EGFR (Cat#4267), p-VEGFR2 (Cat#2478), VEGFR2 (Cat#9698), PDGFRβ (Cat#3169), p-PLCγ1 (Cat#2821), PLCγ1 (Cat#5690), p-GAB1 (Cat#12745), GAB1 (Cat#3232), p-SHC (Cat#2434), SHC (Cat#2432), p-AKT (Cat#4060), AKT (Cat#9272), p-ERK1/2 (Cat#4370), ERK1/2 (Cat#4695), p-STAT3 (Cat#4113), STAT3 (Cat#9139), GRB2 (Cat#3972), GST (Cat#2642) were purchased from Cell Signaling Technology (Danvers, MA, USA). Antibodies against pY (Cat#EPR16871), p-PDGFRβ (Cat#ab248657), p-FGFR1 (Cat#ab59194), α-SMA (Cat#ab124964), pS/pT (Cat#ab117253) and IgG (Cat#EPR25A and Cat#ab37415) were purchased from Abcam (Cambridge, MA, USA). Bleomycin, nintedanib and PHPS1 were purchased from Targetmol (Boston, MA, USA).

### Plasmids and protein purification

Genes encoding the human wild type and triple-mutant Src SH2 domains were subcloned into pGEX-4T3 vector for expressing GST SH2 WT/TrM proteins. Based on pGEX-4T3-alone or pGEX-4T3-SH2 WT/TrM, recombinant plasmids were constructed for expressing GST-(Arg)9, GST-SH2 WT-(Arg)9 and GST-SH2 TrM-(Arg)9. All amplified products were purified using the Gel Band Purification Kit (Vazyme, Nanjing, China) and then were reacted with the linearized pGEX-4T3. The experiment was performed using the One Step Cloning Kit (Vazyme). *E. coli* BL21 (DE3) containing the plasmids above was grown in LB with 100 μg/mL ampicillin at 37 °C. The expression of GST fusion proteins was induced by the addition of isopropyl β-D-thiogalactoside (0.5 mM) and then incubated at 20 °C for 18 h. The protein lysis buffer contains 20 mM Tris-HCl (pH = 7.0), 50 mM NaCl, 0.5 mM EDTA, 1 mM dithiothreitol (DTT), 1 mM cocktail and 1 mM PMSF. GST fusion proteins were purified from bacterial cell lysates with glutathione-agarose beads. The protein concentration was quantified with BCA Protein Assay Kit and protein purity was confirmed by SDS-PAGE using Coomassie brilliant blue staining.

### Isolation of primary lung fibroblasts from human and mice

Primary lung fibroblasts from IPF patients (CCL-191) were obtained from American Type Culture Collection (ATCC, Manassas, VA, USA). The cell culture was performed according to the handling procedure of ATCC. Normal lung fibroblasts were isolated from healthy lung tissue of patients with lung adenocarcinoma (Table S1). All the samples were away from cancerous tissues. Primary lung fibroblasts from control or BLM-treated mice were isolated as previous report 22. Under sterile conditions, human normal lung tissues and adult mouse lung from saline or BLM treated groups were scissored. We isolated the shallow surface tissues of lung to avoid vessels and bronchus. The tissues were minced into 5 mm^3^ pieces, and then placed in 75 cm^2^ cell culture flasks. Ham's F12K medium (Gibco, Grand Island, NY, USA) with 10% fetal bovine serum (FBS) (Gibco) was added into the flasks 2 h later to prevent tissue blocks floating. After 3 days, when the cells isolated from tissues and reached 80% confluence, subculturing procedure was performed. Besides, cells were incubated with antibody against vimentin to determine the mesenchymal phenotype. All cells were cultured under 5% CO_2_ at 37 °C and used for all experiments within 3-5 passages. Cells were synchronized for 6 h with 2% FBS F12K medium before directly being used for the subsequent experiments.

### Isolation of primary epithelial cells, endothelial cells and leukocytes

The whole mouse lungs were perfused with saline through the right ventricle of heart to eliminate blood cells. The pulmonary lobes were separated into small pieces and washed in PBS on ice. Then the lung pieces were incubated in PBS containing 2 mg/mL collagenase (Sigma, St. Louis, MO, USA) and DNase I 2 μg/mL (Sigma) for 45 min at 37 °C and cell suspension was passed through 70 μm strainers. After centrifugation at 300 g for 10 min at 4 °C, cell pellets were resuspended in ack lysis buffer to remove red blood cells. Then cells were incubated with antibodies including PE anti-mouse EpCAM (Biolegend, San Diego, CA, USA, Cat#118205), FITC anti-mouse CD45 (Biolegend, Cat#103108) and APC anti-mouse CD31 (Biolegend, Cat#102509) for 1 h on ice. After washed with PBS, cells were sorted by FACS.

### Western blot analysis

Proteins of cell lysates and tissues were denatured and electrophoresed on SDS-PAGE gel. The dilution concentrations of primary antibodies were 1:1000 (pY), 1:1000 (pS/pT) 1:2000 (FN), 1:1000 (COL1A1), 1:2000 (α-SMA), 1:1000 (p-EGFR), 1:1000 (EGFR), 1:1000 (p-VEGFR2), 1:1000 (VEGFR2), 1:1000 (p-PDGFRβ), 1:1000 (PDGFRβ), 1:1000 (p-FGFR1), 1:1000 (FGFR1), 1:1000 (p-PLCγ1), 1:1000 (PLCγ1), 1:1000 (p-GAB1), 1:1000 (GAB1), 1:1000 (p-SHC), 1:1000 (SHC), 1:1000 (p-AKT), 1:2000 (AKT), 1:1000 (p-STAT3), 1:1000 (STAT3), 1:1000 (p-ERK1/2), 1:2000 (ERK1/2), 1:2000 (GST), 1:10000 (GAPDH). After primary antibodies being incubated overnight at 4 °C, HRP-conjugated secondary antibodies (ABclonal, Woburn, MA, USA) were incubated at room temperature for 1 h (dilution 1:3000). Images were developed by ChemiDoc MP Image and Image-Pro Plus software was used for densitometric quantification.

### CCK-8 assay

Lung fibroblasts collected in logarithmic phase were plated into 96-well plates (6×10^3^ cells/well). According to the protocol of CCK-8 (Targetmol), 10 μL of CCK-8 solution diluted in 100 μL of medium replaced the culture medium. The cells were incubated at 37 °C for 2 h and the absorbance was detected at 450 nm wavelength.

### EdU assay

EdU kit (Abbkine Scientific, Wuhan, China) was employed according to the manufacturer's instruction. Lung fibroblasts were seeded in 24-well plates and then incubated with 10 μM EdU for 2 h. The fibroblasts were fixed with 4% formaldehyde for 20 min at 37 °C, followed by permeabilization in 0.5% Triton X-100 for 15 min. Then 100 μL Click-iT was added to each well and incubated for 30 min in dark at room temperature. After being washed with PBS, the nuclei were stained with DAPI for 10 min and images were captured by fluorescence microscope (Olympus, Tokyo, Japan).

### Wound healing assay

Cells were seeded into 6-well plates and then scratched with the tip of a p-10 pipette to uniform cell-free zone in each well. The scratched cells were removed by PBS and then incubated with indicated conditions. Microscopic images were taken with a digital camera under inverted microscope. The unrecovered area was measured by using Image J software and expressed as a percentage of the initial scratched area.

### Transwell migration assay

8 μm pore transwell inserts were purchased from Corning Inc (Corning, NY, USA). Cells were seeded into the upper chamber of inserts with FBS-free medium, and 10% FBS medium was added to lower chamber. Different stimuli were also added to upper chamber. When the experiment finished, transwell inserts were fixed with 4% triformol for 20 min and then stained with crystal violet for 30 min. Then the upper cells were wiped off, and cells on the lower surface were captured by digital camera of inverted microscope. Image J software was used for cell counting.

### Transmission electron microscopy (TEM) analysis

Lung fibroblasts were fixed in 2.5% glutaraldehyde with 0.1 M phosphate (pH 7.2) for 2 h, followed by the fixation with 1% osmium tetroxide for 1 h. 10 nm sections were sliced and stained with 2% uranyl acetate after dehydration in graded ethanol. JEM1230 transmission electron microscope (JEOL, Tokyo, Japan) was performed to determine the lipid drops in lung fibroblasts.

### Nile red staining

Cells were fixed with 4% triformol for 20 min and then incubated with Nile red (dilution 1:1000) (MedChemExpress, Monmouth Junction, NJ, USA) for 15 min. After three times washing of PBS, DAPI was dyed for 10 min. Images were captured by fluorescence microscope.

### Immunofluorescence of lung fibroblasts

To directly indicate the changes of protein expression and localization of different proteins, immunofluorescence was used. Cells were seeded on slides. After different treatments, cells were incubated with antibodies against pY (dilution 1:50), α-SMA (dilution1:100), GST (dilution 1:50), EGFR (dilution 1:50) and SHC (dilution 1:50) overnight at 4 °C. According to different species of the primary antibodies, Cy3-or FITC-labeled rabbit or mouse secondary antibodies (dilution 1:200) were incubated for 1 h at room temperature in the dark. At last, DAPI was used for nucleus staining for 10 min. Zeiss-LSM800 confocal laser scanning microscope (Carl Zeiss AG, Oberkochen, German) was used for observation and picture collection.

### Immunoprecipitation

Cells were lysed on ice in lysis buffer (1% NP-40, 50 mM Tris-HCl (pH = 7.4), 150 mM NaCl, 2 mM EDTA, 50 mM NaF, 10% glycerol, and the complete protease inhibitor cocktail). The supernatant was gathered after centrifugation at 12,000 g for 15 min at 4 °C. Protein concentrations were quantified by BCA method. The lysate was cleaned with an appropriate pre-immune serum and protein A/G agarose (Thermo Fisher, Waltham, MA, USA), immunoprecipitation was then performed using indicated antibodies. Protein expressions were determined by western blot as described above.

### GST pull down assay

GST fusion proteins were expressed in *E. coli* BL21 (DE3). Cells were treated with phosphatase inhibitor sodium pervanadate (0.5 mM) for 10 min at 37 °C before harvesting. Then, cells were lysed in ice-cold lysis buffer (0.5% NP-40, 50 mM Hepes (pH = 7.4), 1 mM magnesium chloride, 150 mM KCl, and the complete protease inhibitor cocktail). Glutathione Sepharose beads (GE Healthcare, Chicago, IL, USA) were used for the GST pull down assay. Protein concentrations were quantified by BCA method. Pull down was performed by incubating GST-tagged proteins with cell lysates for 3 h, the beads were then washed with lysis buffer for three times at 4 °C. Proteins bound to GST beads were resolved on SDS-polyacrylamide gel electrophoresis and identified by western blot as described above.

### Live and dead cell staining assay

Live and dead cells double staining kit was purchased from Abbkine. According to manufacturer's instruction, we seeded cells into 24-well plates. 0.5 mL staining solution was added to plates and incubated with cells at 37 °C for 30 min in the dark. Images were captured by fluorescence microscope.

### Animal models

In this study, all animals were specific-pathogen-free (SPF) 8 weeks old male C57BL/6J mice and housed under standard conditions with free access to water and food. The research protocol was under the auspices of the Institutional Review Board of Tongji Medical College, Huazhong University of Science and Technology. All animal experiments were performed in accordance with the Guide for the Care and Use of Laboratory Animals and approved by the Institutional Animal Care and Use Committee (IACUC) of Tongji Medical College, Huazhong University of Science and Technology.

Single dose intratracheal BLM model: Mice were treated with 50 μL BLM (2 U/kg) or 0.9% NaCl by intratracheal injection at day 1, and intratracheal injection was performed with PBS, GST-(Arg)9, GST-SH2 WT-(Arg)9, GST-SH2 TrM-(Arg)9, SH2 TrM-(Arg)9 or nintedanib in BLM groups at day 14 and 17. All mice were sacrificed at day 21.

Repetitive intratracheal BLM model: According to previous report 23, mice were intratracheally injected with 50 μL BLM (1 U/kg) or 0.9% NaCl every two weeks for 16 weeks. After final BLM injection, mice were next intratracheally injected with GST-(Arg)9, GST-SH2 WT-(Arg)9, GST-SH2 TrM-(Arg)9 or SH2 TrM-(Arg)9 in BLM groups and PBS in saline groups. The treatments lasted twice a week for another two weeks. All mice were sacrificed at the end of week 18.

Intraperitoneal BLM administration model: As our previous report 24, mice were intraperitoneally injected with BLM (40 U/kg) at day 1, 5, 8, 11 and 15. At day 24, 28, 34 and 36, PBS, GST-(Arg)9, GST-SH2 WT-(Arg)9, GST-SH2 TrM-(Arg)9 or SH2 TrM-(Arg)9 were intratracheally administrated in BLM groups, and in saline group, PBS was intratracheally injected as well. All the mice were sacrificed at day 40.

### MicroCT scanning

Mice were scanned using high-resolution microCT scanner SkyScan 1176 (Bruker, Kontich, Belgium). After preliminary experiments, we chose 18 μm resolution ratio and Al 1 μm filter as scanning condition. The scanning voltage was 55 kVp and current was 355 μA. CTvox was used to perform image reconstruction. The lung volume was also determined by 3D reconstruction by CTvox.

### Measurement of respiratory mechanics

To perform respiratory mechanics analysis, mice were anesthetized using Nembutal solution of pentobarbital (50 mg/kg). After performing a tracheotomy and cannulating the trachea, mice were connected to Forced Manoeuvres System (CRFM100, EMMS, Bordon, UK). Mice had autonomous respiration and were monitored in whole-body plethysmograph with a pneumotachograph connected to transducer. The parameters, including compliance, elastance, resistance, and functional residual capacity (FRC), inspiratory volume (IC), residual volume (RV), total lung capacity (TLC) and peak expiratory flow were obtained.

### Hematoxylin-eosin (HE) and Masson's trichrome staining of mouse lung tissues

The fixed lungs were embedded in paraffin. 5 μm thick sections were stained with hematoxylin-eosin or Masson's trichrome for assessing the structural change, inflammation and collagen deposition. Images were captured by digital camera of upright microscope. Image analysis was carried out through Image J software.

### Immunohistochemistry staining

Lung sections were embedded, sectioned and deparaffinized followed by antigen retrieval after fixation. Sections were incubated with primary antibodies α-SMA (dilution 1:1000), pY (dilution 1:400) and pS/pT (dilution 1:200) overnight at 4 °C. On next day, sections were washed and then incubated with biotinylated secondary antibodies for 20 min. The slides were developed with DAB working solution, followed by counter staining with hematoxylin and mounting. The slides were scanned by microscope connected with a digital camera.

### Immunofluorescence staining

Lung sections were embedded, sectioned and deparaffinized followed by antigen retrieval after fixation. Sections were incubated with antibodies against pY (dilution 1:50), pS/pT (dilution 1:50), α-SMA (dilution1:100), CD45 (dilution 1:100), CD31 (dilution 1:50), EpCAM (dilution 1:50) and GST (dilution 1:200) overnight at 4 °C and then FITC-conjugated goat anti-mouse antibody (dilution 1:200) and Cy3-conjugated goat anti-rabbit antibody (dilution 1:200) were incubated together for 1 h. The nuclei were stained with DAPI for 10 min. The pictures were taken by confocal laser scanning microscope.

### Hydroxyproline assay

The hydroxyproline level was measured using a commercial kit from Nanjing Jiancheng Crop (Nanjing, China). Briefly, precisely weighed lung homogenates were hydrolyzed with 5% sodium hydroxide, mixed with indicative reagent. When the mixture became flavescent, PH value was adjusted to 6.0-6.8. After centrifugation, the contents of supernatants were measured at 560 nm by spectrophotometer. The hydroxyproline content was calculated by the standard curve.

### Soluble collagen content assay

Sircol soluble collagen assay was purchased from Biocolor (Newtownabbey, Northern Ireland). Lung homogenates were incubated with pepsin at room temperature overnight. 50 μL of collagen isolation and concentration reagent from the kit were added and incubated overnight at 4 °C. Different samples were centrifuged at 12000 rpm for 15 min and supernatants were abandoned. 200 μL of sircol dye reagents were added and incubated at room temperature for 30 min and centrifuged at 12000 rpm for 15 min. Then 200 μL of pre-cooling acid-salt wash was layered over the collagen-dye pellet, mixed and centrifuged at 12000 rpm for 15 min. The supernatants were abandoned and 200 μL of alkali reagent was added to the collagen bound dye. The samples were measured at 550 nm by spectrophotometer. The soluble collagen content was calculated by the standard curve.

### Myeloperoxidase (MPO) assay

MPO activity in lung tissues was measured with MPO assay kit (Nanjing Jiancheng Crop). Lung tissues were accurately weighed, and then homogenized buffer was added. After being mixed well, test tubes were incubated in water bath at 37 °C for 15 min and then centrifuged at 12000 rpm. MPO activity was determined in 10 μL of samples by measuring activity over 120 min period. All samples were measured at 460 nm with a colorimetric microplate reader.

### BALF cell counting and sorting

We used 0.5 mL saline to lavage lungs three times and collected the fluids into one tube. 50 μL alveolar lavage fluid was used for counting by a hemocytometer. Besides, cells in BALF were stained using Giemsa staining. Depending on the different color of leukocytes, we further calculated the proportion of different kinds of cells. Image J software was used for cell counting.

### ELISA assay

The amounts of IL-1β, IL-6, IL-10 and TNF-α in BALF of mice were measured by using mouse ELISA kits from R&D Systems (Minneapolis, MN, USA). The procedures were performed according to the manufacturer's instructions.

### Alanine aminotransferase (ALT), aspartate aminotransferase (AST) and lactic dehydrogenase (LDH) assay

We collected blood via fundus venous plexus of mice. After whole blood centrifuged, upper serums were used to detect the content of ALT, AST and LDH. We used ALT, AST and LDH assay kits of Nanjing Jiancheng Crop and procedures were performed according to the manufacturer's instructions.

### Extraction of RNA from lung tissues and RT-qPCR

TRIzol reagent (Vazyme) was used for RNA extraction from lung tissues. RNA levels of *COL1A2*, *COL3A1*, *COL5A1*, *COL5A2*, *MMP-7*, *MMP-9* and *TIMP-1*, were determined by RT-qPCR. The primers were as follows, *COL1A2*: forward 5'-CAGAACATCACCTACCACTGCAA-3'; reverse 5'-TTCAACATCGTTGGAACCCTG-3'; *COL3A1*: forward 5'-AGGCTGAAGGAAACAGCAAA-3'; reverse 5'-TAGTCTCATTGCCTTGCGTG-3'; *COL5A1*: forward 5'-CGTGGGTTTGATGGTCTGG-3'; reverse 5'-GGCCATCCATACCCGTTAC-3'; *COL5A2*: forward 5'-GAAAGGGACAAAAAGGAGAACCAG-3'; reverse 5'-TTCACCTGTATTCCCATTTCTTCC-3'; *MMP-7*: forward 5'-CAGGAAGCTGGAGATGTGAGC-3'; reverse 5'-GAGAGTTTTCCAGTCATGGGC-3'; *MMP-9*: forward 5'-CATTCGCGTGGATAAGGAGT-3'; reverse 5'-CACTGCAGGAGGTCGTAGG-3'; *TIMP-1* forward 5'-GATATGCCCACAAGTCCCAGAACC-3'; reverse 5'-GCACACCCCACAGCCAGCACTAT-3'.

### Statistical analysis

Expression profiling by array and RNA-seq data were reanalyzed by R 4.0.2. All other experimental results were analyzed using GraphPad Prim software (version 8.0.2). Data were represented as the mean ± SEM. Statistical analyses between two groups were compared using two-sided Student's t test and between multiple groups were compared using one-way ANOVA test. Correlation test was performed by Pearson's Correlation Tests. Log-rank (Mantel-Cox) test was used for survival analysis P < 0.05 was accepted as statistical significance.

## Results

### Tyrosine phosphorylation mediated signaling pathways were associated with IPF

**Figure 1:**
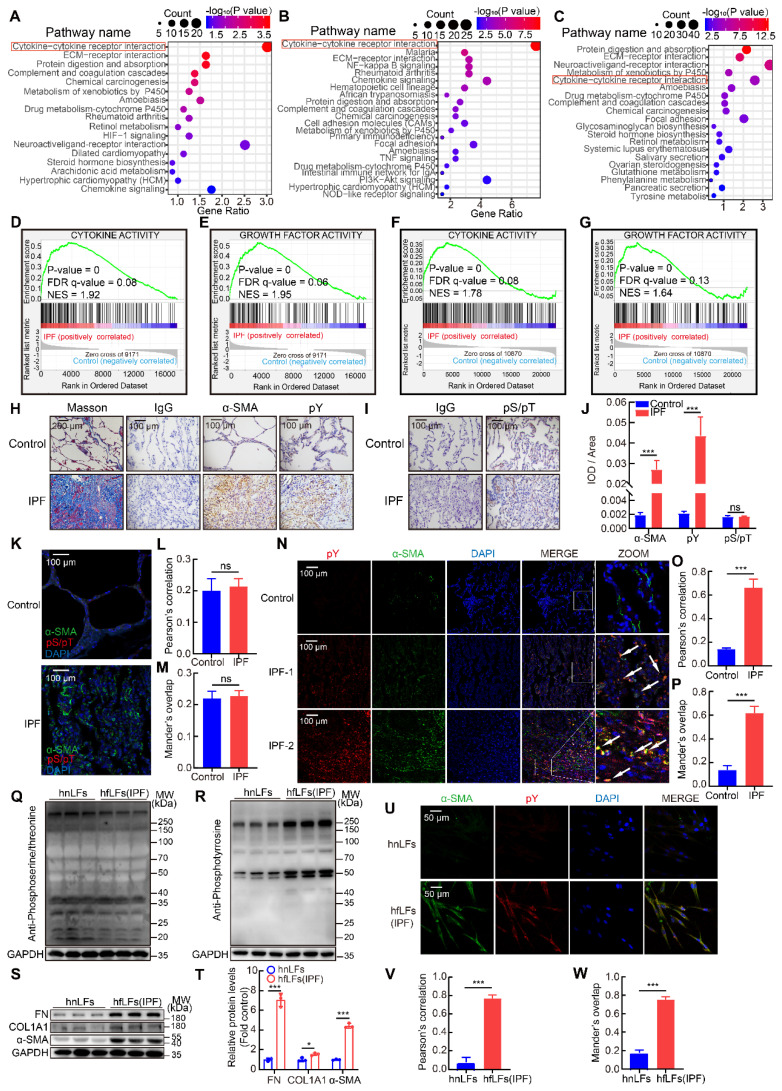
** High pY levels induced by activation of cytokine and growth factor pathways were found in IPF lung tissues and fibroblasts. A**, KEGG pathway enrichment analysis in lung tissues from IPF patients (n = 122) and control (n = 91) in dataset GSE47460. **B**, KEGG pathway enrichment analysis in lung tissues from IPF patients (n = 40) and control (n = 8) in dataset GSE53845. **C**, KEGG pathway enrichment analysis in lung tissues from IPF patients (n = 20) and control (n = 19) in dataset GSE92592. **D-E**, GSEA analysis in IPF dataset GSE47460. **F-G**, GSEA analysis in IPF dataset GSE71351. **H**, Representative images of Masson's trichrome staining, α-SMA and pY immunostaining of lung tissue sections from IPF patients and healthy control. IgG was an isotype control for α-SMA and pY. **I**, Representative images of pS/pT immunostaining of lung tissue sections from IPF patients and healthy control. IgG was an isotype control for pS/pT. **J**, Quantification analysis of immunostaining with α-SMA, pY and pS/T (n = 3). ^***^P < 0.001. **K**, Representative fluorescence images of pS/pT of lung tissue sections from different IPF patients and healthy control. **L-M**, Co-location analysis of pS/pT and α-SMA (n = 3). **N**, Representative fluorescence images of pY and α-SMA of lung tissue sections from IPF patients and healthy control. The boxed regions were revealed at higher magnification and arrows indicated overlap of pY and α-SMA in lung tissues. **O-P**, Co-location analysis of pY and α-SMA (n = 3). ^***^P < 0.001. **Q-R**, Western blot of pS/T and pY in hnLFs and hfLFs. GAPDH was used as a loading control. **S**, Western blot of FN, COL1A1 and α-SMA in lung fibroblasts from hnLFs and hfLFs. GAPDH was used as a loading control. **T**, Quantification of FN, COL1A1 and α-SMA protein levels in hnLFs and hfLFs (n = 3). ^*^P < 0.05, ^***^P < 0.001. **U**, Representative fluorescence images of α-SMA and pY in hnLFs and hfLFs. **V-W**, Co-expression analysis of pS/pT and α-SMA (n = 3). ^***^P < 0.001.

To confirm the protein contribution involved in tyrosine phosphorylation mediated signaling pathways in IPF patients, we performed bioinformatics analysis from IPF database. Due to tyrosine phosphorylation induced by the combination of cytokines and growth factors with RTKs, we firstly tried to explore the possible connection between IPF and cytokines as well as growth factors. We analyzed three datasets of IPF patients and relevant control. Through the analysis of gene expression profiling of GSE47460, GSE53845 and mRNA sequencing of dataset GSE92592, we found that cytokine-cytokine receptor interaction pathway was significantly correlated with IPF in KEGG pathway enrichment analysis (Figure 1A-C). Meanwhile, GSEA analysis was used for GSE47460 and the data displayed the activation of multiple cytokine and growth factor pathways were robustly correlated with IPF (Figure 1D-E). Besides, the impaired lung function such as DL_co_ was significantly correlated with cytokine-cytokine receptor interaction pathway in IPF patients (Figure S1A). Then we further investigated the top five differential expression genes in normal and IPF lung tissues from dataset GSE47460 and found that two genes (*IL13RA2* and *IGFL2*) were involved in cytokines and growth factors mediated pathways (Figure S1B-C). The two genes were also strongly correlated with decline of lung function included DL_co_, FEV1 pre-BD, FVC pre-BD, FEV1 post-BD and FVC post-BD (Figure S1D-F). They were both positively correlated with other fibrosis markers (*COL1A2*, *COL3A1*, *COL5A1*, *COL5A2*, *ACTA2*, *MMP7*, *MMP9* and *TIPM1*) (Figure S1G). Moreover, in database GSE71351 of IPF lung fibroblasts, GSEA analysis also confirmed cytokine and growth factor pathways were activated (Figure 1F-G and Figure S1H-K).

Evoked by cytokines, cytokines receptors would be activated by autophosphorylation and transmit phosphorylation signals downstream, mainly included pY, phosphoserine (pS) and phosphothreonine (pT) 25. Based on our analysis of IPF databases, we tried to further confirm cytokine-cytokine receptor interaction induced phosphorylation in clinical specimens of IPF patients. Using immunostaining, we found that pY levels were elevated in IPF lung tissues than those in healthy control, along with higher expressions of a canonical myofibroblasts marker, alpha smooth muscle actin (α-SMA), while pS/pT levels were not significantly changed (Figure 1H-J). In addition, STAT3, AKT, and ERK1/2 signaling pathways, which located downstream of pY-mediated pathways were accordantly activated in IPF (Figure S1L-M). Moreover, pS/pT did not co-localize with α-SMA (Figure 1K-M) but pY and α-SMA exhibited strong co-localization in fibrotic lung tissues than that in health control, which suggested that the pY levels were significantly increased in myofibroblasts (Figure 1N-P). Besides, pY showed moderate expression in epithelial cells, leukocytes, and endothelial cells in lung of IPF (Figure S1N-P). Next, we evaluated the expression of pS/pT, pY and fibrotic proteins in lung fibroblasts from IPF patients (hfLfs) and control subjects (hnLfs). Compared to hnLfs, the expression of pS/pT in hfLfs showed no difference (Figure 1Q), while high levels of pY, fibronectin (FN), collagen-Iα1 (COL1A1) and α-SMA were found in hfLfs (Figure 1R-T). Immunofluorescence also showed that the levels of pY and α-SMA in hfLfs were remarkably increased compared to hnLfs (Figure 1U-W).

### Fluctuation of pY levels were associated with BLM-induced pulmonary fibrosis in mice

**Figure 2:**
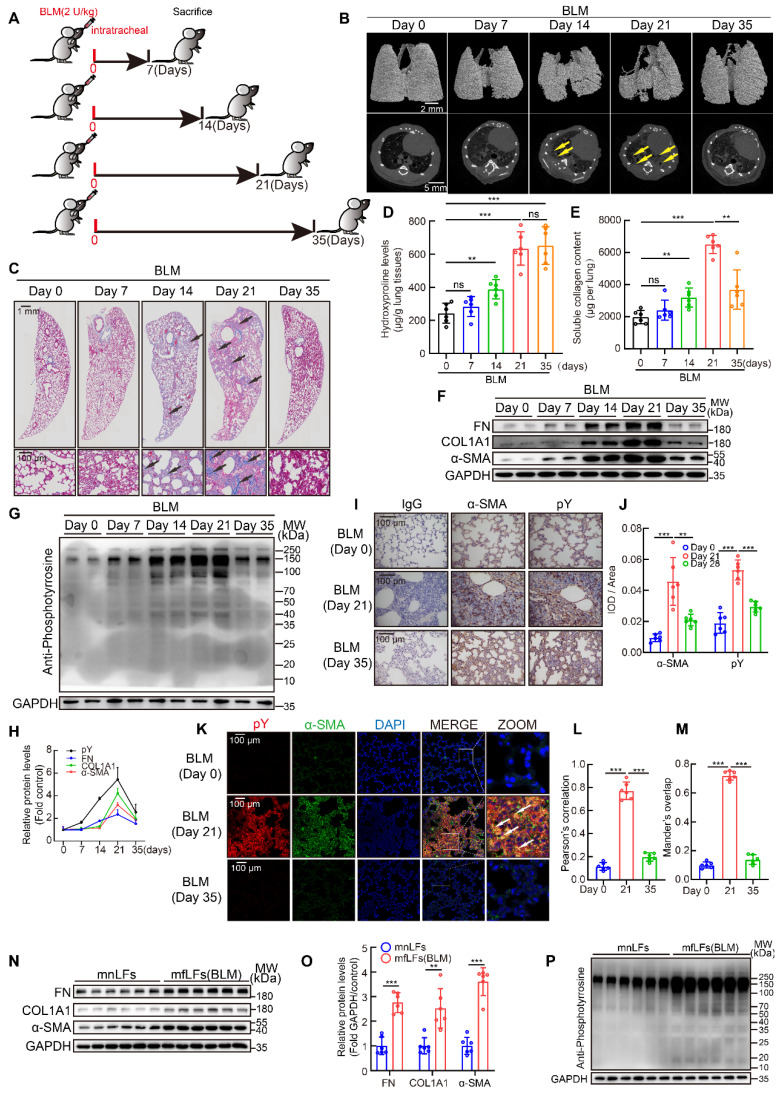
** Fluctuation of pY levels was associated with BLM-induced pulmonary fibrosis in mice. A**, Schematic of BLM-induced pulmonary fibrosis for 35 days in mice. **B**, Representative microCT reconstitution images of 3D and cross sections of lungs. Arrows showed the fibrosis foci which resulted in serious parenchymal destruction and volume reduction of lungs. **C**, Masson's trichrome staining after BLM administration. Images showed the panoramic and partial view of lungs. Arrows pointed to the collagen deposition. **D**, Lung hydroxyproline content after BLM treated (n = 6). ^**^P < 0.01,^ ***^P < 0.001. **E**, Soluble collagen content after BLM treated (n = 6). ^**^P < 0.01, ^***^P < 0.001. **F**, Western blot of FN, COL1A1 and α-SMA in lung tissues of BLM-treated mice. **G**, Western blot of pY in lung tissues of BLM-treated mice. **H**, The variation trends of FN, COL1A1, α-SMA and pY in the progress of BLM model (n = 6). **I**, Representative images of pY and α-SMA immunostaining of lung tissue sections in saline or BLM group. **J**, Quantification analysis of immunostaining with α-SMA and pY (n = 6). ^**^P < 0.01,^ ***^P < 0.001. **K**, Representative fluorescence images of pY and α-SMA of lung tissue sections from BLM-treated mice at day 0, 21 and 35. The boxed regions were revealed at higher magnification and arrows indicate overlap of pY and α-SMA in lung tissues. **L**-**M**, Co-location analysis of pY and α-SMA (n = 6). ^***^P < 0.001. **N**, Western blot of FN, COL1A1 and α-SMA in mnLFs and mfLFs (n = 6). **O**, Quantification of FN, COL1A1 and α-SMA protein levels in mnLFs and mfLFs (n = 6). ^**^P < 0.01,^ ***^P < 0.001. **P**, Western blot of pY in mnLFs and mfLFs (n = 6).

BLM mouse model is the most extensively used and best-characterized to duplicate pulmonary fibrosis 26. Although single dose BLM injection could induce pulmonary fibrosis at day 21 in the model, a spontaneous resolution of lung injury after day 28 usually results in fibrosis self-healing 27. To further evaluate the relation between pY levels and pulmonary fibrosis *in vivo*, an intratracheal infusion of single dose BLM model for 35 days was employed in mice. C57BL/6 mice were intratracheally injected with a single dose of BLM (2 U/kg) and lung tissues were collected at day 7, 14, 21 and 35 (Figure 2A). MicroCT scan (Figure 2B) and Masson's trichrome staining (Figure 2C) revealed that the fibrosis foci gradually appeared and quickly expanded from day 7 to day 21. But from day 21 to day 35, fibrosis foci disappeared and lungs almost restored to normal structure. Hydroxyproline and soluble collagen levels in lung tissues were also increased at day 14 and day 21, but soluble collagen reduced while hydroxyproline also kept in high level at day 35 (Figure 2D-E). Meanwhile, the expressions of FN, COL1A1 and α-SMA in lung tissues were significantly increased at day 21 and decreased at day 35 (Figure 2F). All results above suggested that fibrosis appeared and became progressively worse from day 0 to 21, whereas gradually self-healing from day 21 to 35. Moreover, the pY levels in lung tissues were also up-regulated from day 0 to 21 and reduced from day 21 to day 35 (Figure 2G). The change of pY levels was paralleled with fibrosis markers (Figure 2H). We then detected pY levels in lung sections and lung fibroblasts from BLM-treated mice to further investigate the relationship between myofibroblasts activation and high pY levels. Immunohistochemical showed that pY and α-SMA were highly expressed in fibrotic area of BLM-treated mice at day 21, but down-regulated at day 35 (Figure 2I-J). Immunofluorescence staining also confirmed that myofibroblasts expressed high pY levels at day 21, but decreased pY levels at day 35 after BLM-treated (Figure 2K-M). Isolated pulmonary fibroblasts from BLM-treated mice of 21 days (mfLfs) expressed enhance levels of FN, COL1A1 and α-SMA than fibroblasts from control mice (mnfLfs) (Figure 2N-O). As expected, pY levels of mfLfs were also significantly higher than those of mnfLfs (Figure 2P). These data suggested that elevated pY levels of lung fibroblasts may be associated with BLM-induced pulmonary fibrosis.

### High pY levels might affect the proliferation, migration and differentiation of lung fibroblasts

**Figure 3:**
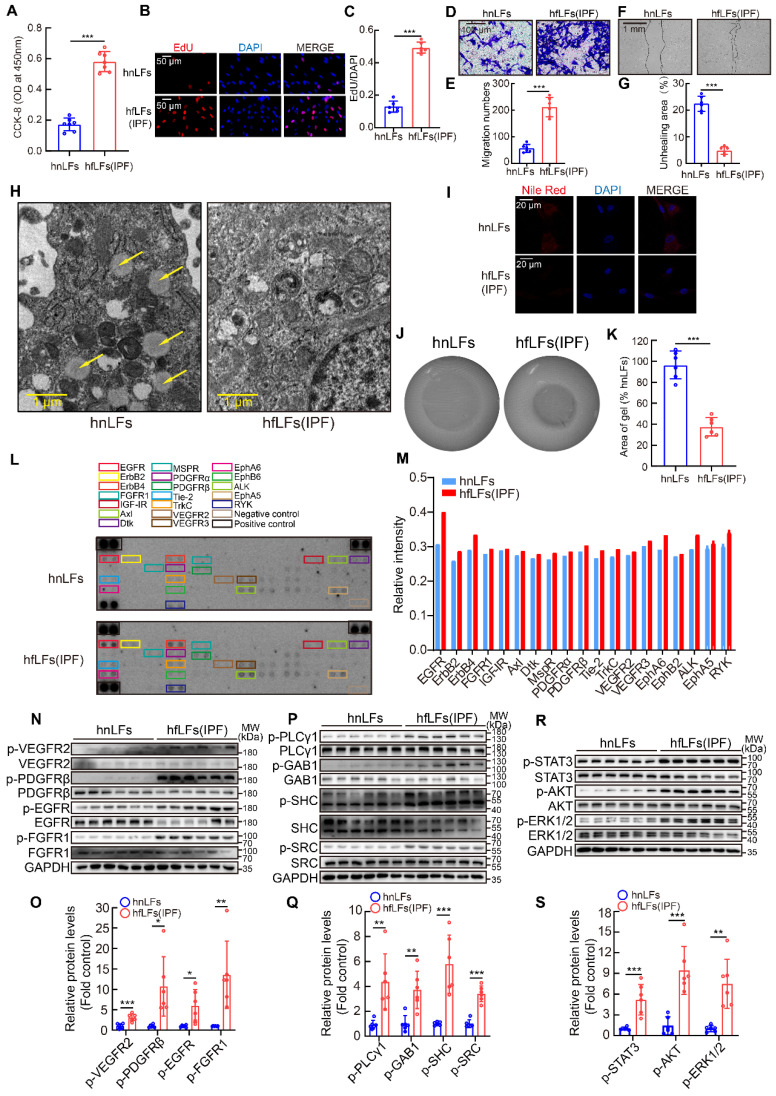
** High pY levels might affect the proliferation, migration and differentiation of lung fibroblasts. A**, Changes in cell proliferation between hnLFs and hfLFs determined by CCK-8 kit (n = 7). ^***^P < 0.001. **B-C**, Assessing cell proliferation by EdU assay between hnLFs and hfLFs (n = 6).^ ***^P < 0.001. **D-G**, Comparison of migration between hnLFs and hfLFs (n = 6).^ ***^P < 0.001. **H**, Representative images of hnLFs and hfLFs under TEM. Arrows pointed to the lipid droplets in cytoplasm. **I**, Representative fluorescence images of Nile Red in hnLFs and hfLFs.** J-K**, The contractility of hnLFs and hfLFs in 3D collagen matrices (n = 6). ^***^P < 0.001. **L**, Phospho-RTK array analysis with 200 μg of lysates from hnLFs and hfLFs, which were serum-starved for 6 h. **M**, Quantification of phospho-RTK levels (average of two drops fold average of positive control). **N-O**, Western blot and quantification of p-VEGR2, p-PDGFRβ, p-EGFR and p-FGFR1 in hnLFs and hfLFs (n = 6). ^*^P < 0.05, ^**^P < 0.01, ^***^P < 0.001. **P-Q**, Western blot and quantification of p-PLCγ1, p-GAB1, p-SHC and p-SRC in hnLFs and hfLFs (n = 6). ^**^P < 0.01, ^***^P < 0.001. **R-S**, Western blot and quantification of p-AKT, p-ERK1/2 and p-STAT3 in hnLFs and hfLFs (n = 6). ^**^P < 0.01, ^***^P < 0.001.

Next, we explored whether increased pY levels were related to the proliferation, migration and differentiation of fibroblasts from fibrotic lung tissues *in vitro*. HfLfs and mfLfs displayed markedly elevated proliferation than hnLfs and mnLfs (Figure 3A-C and Figure S2A-C). Transwell and wound-healing assays revealed hfLfs and mfLfs had stronger migration abilities (Figure 3D-G and Figure S2D-G). Decreasing intracellular lipid droplets and increasing contractility of fibroblasts are the indicators of differentiation from fibroblasts to myofibroblasts 28, 29. TEM and Nile red revealed that hfLfs and mfLfs lost lipid droplets compared with hnLfs and mnLfs (Figure 3H-I and Figure S2H-I). The contractility of hfLfs and mfLfs was also higher than control (Figure 3J-K and Figure S2J-K).

To further clarify the relationship between abnormal manifestations of lung fibroblasts and high pY levels, we examined phosphorylation of several specific RTKs, which trigger intracellular signaling cascades through pY-SH2 domain combination after cytokines or growth factors binding. Phospho-RTK array indicated that the pY levels of numerous RTKs in hfLfs were higher than those in hnfLfs, regardless of slightly increase of pY levels of several RTKs in hfLfs (Figure 3L-M). Furthermore, we detected intracellular specific phosphorylation sites of 4 RTKs in hfLfs and hnfLfs, including VEGFR2 (Tyr1175), EGFR (Tyr1068), PDGFRβ (Tyr1021) and FGFR1 (Tyr654), which were involved in fibrotic diseases 30-33. We found higher pY levels in hfLfs than those in hnfLfs (Figure 3N-O). In mfLfs, we also discovered elevated pY levels at above 4 sites (Figure S2L-M). Phospholipase Cγ1 (PLCγ1), Grb associated binder 1 (GAB1), Src homology 2 domain containing transforming protein 1 (SHC) and SRC act as adaptor proteins in pY-mediated signaling pathways downstream of RTKs. Accordingly, higher pY levels were also found in hfLfs and mfLfs (Figure 3P-Q and Figure S2N-O). Furthermore, three important downstream cytokines and growth factors signaling pathways, PI3K/AKT, MAPK/ERK, and JAK/STAT pathways, were significantly activated (Figure 3R-S and Figure S2P-Q).

These results enlightened that high pY levels would be correlated with excessive proliferation, migration and differentiation of lung fibroblasts, of which the activation of multiple signaling pathways might be involved in the process.

### SH2 superbinder suppressed the proliferation, migration and differentiation of lung fibroblasts* in vitro*

**Figure 4:**
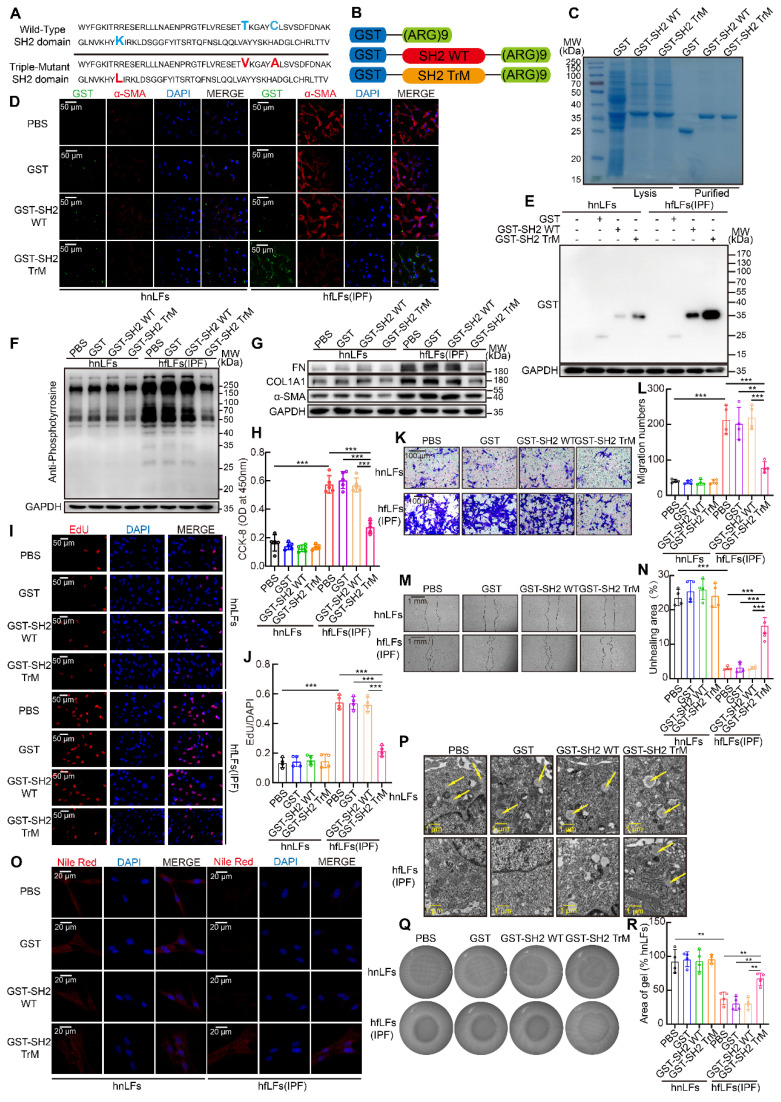
** SH2 superbinder suppressed proliferation, migration and differentiation of lung fibroblasts *in vitro.* A**, AA sequence of SH2 WT and SH2 TrM. **B**, Recombinant protein structure of GST-(Arg)9, GST-SH2 WT-(Arg)9 or GST-SH2 TrM-(Arg)9. **C**, Identification of recombinant protein expression and purification by Coomassie blue staining. According to concentration gradient and time gradient effect, we treated hnLFs and hfLFs with 1 μM GST, GST-SH2 WT or GST-SH2 TrM for 24 h. **D**, Representative fluorescence images of GST, GST-SH2 WT or GST-SH2 TrM entrance the cells. **E**, Western blot of GST tag in hnLFs and hfLFs after GST, GST-SH2 WT or GST-SH2 TrM incubation. **F-G**, Western blot of pY (**F**) and FN, COL1A1, α-SMA (**G**) in hnLFs and hfLFs after treatment of GST, GST-SH2 WT or GST-SH2 TrM. **H-J**, Changes in cell proliferation between hnLFs and hfLFs after GST, GST-SH2 WT or GST-SH2 TrM incubation determined by CCK-8 and EdU assay (n = 5 or 4).^ ***^P < 0.001. **K-N**, Comparison of migration between hnLFs and hfLFs after GST, GST-SH2 WT or GST-SH2 TrM treatment (n = 4).^ ***^P < 0.001. **O-P**, Differentiation measured by content of lipid droplets through TEM and Nile red staining after GST, GST-SH2 WT or GST-SH2 TrM treatment. Arrows pointed to the lipid droplets in cytoplasm. **Q-R**, The contractility of hnLFs and hfLFs after GST, GST-SH2 WT or GST-SH2 TrM treatment (n = 4).^ **^P < 0.01.

SH2 superbinder has three AA mutations compared with wild type SH2 domain of SRC which leads to a stronger binding ability to pY (Figure 4A). Furthermore, to facilitate the penetration of SH2 superbinder through cell membranes, we modified SH2 superbinder with (Arg)9 (Figure 4B). Next, we obtained the purified GST fusion proteins of GST-(Arg)9 (GST for short), GST-SH2 WT-(Arg)9 (GST-SH2 WT for short) and GST-SH2 TrM-(Arg)9 (GST-SH2 TrM for short) (Figure 4C). Due to the up-regulated pY levels in hfLfs, more GST-SH2 TrM passed through cell membranes and maintained in the cells by competitively capturing pY (Figure 4D-E). But in hnLfs, GST-SH2 TrM barely bound to pY or maintained in the cells because of lower pY levels. So, GST-SH2 TrM had little toxic effect on hnLfs and did not change the survival of hnLfs (Figure S3A). Taken advantage of the stronger competitive combination, GST-SH2 TrM, but not GST-SH2 WT, could decrease pY levels and further reduce the expression of FN, COL1A1 and α-SMA (Figure 4F-G). The repression was in a dose- and time- dependent manner (Figure S3B-E). The proliferation (Figure 4H-J) and migration (Figure 4K-N) of lung fibroblasts were also suppressed by GST-SH2 TrM in hfLfs and so did the dose- and time-dependent inhibition of GST-SH2 TrM (Figure S3F-M). Meanwhile, GST-SH2 TrM exhibited the ability to reverse the loss of lipid droplets in hfLfs (Figure 4O-P and Figure S3N-O), and repressed fibroblasts differentiation into myofibroblasts as well as decreased contractility of hfLfs (Figure 4Q-R and Figure S3P-S). To further explore the effect of increased pY levels on proliferation, migration and differentiation of fibroblasts, we treated hnLfs with PHPS1 (an inhibitor of SHP-2), which could inhibit dephosphorylation of tyrosine and has been proved to induce differentiation of fibroblasts to myofibroblasts in lung 34. After treated with PHPS1, we found that pY levels were significantly increased in hnLfs (Figure S4A). We also confirmed that PHPS1 induced proliferation, migration, differentiation and ECM production of hnLfs could be restrained by GST-SH2 TrM (Figure S4B-L).

In mfLfs, GST-SH2 TrM also reduced pY, FN, COL1A1 and α-SMA levels (Figure S5A-B). Aberrantly proliferation and migration capacities of mfLfs were suppressed by GST-SH2 TrM (Figure S5C-I). Lipid droplets recurred in the cytoplasm of mfLfs after GST-SH2 TrM treated (Figure S5J-K). The contractility was also reversed after GST-SH2 TrM administration (Figure S5L-M).

### SH2 superbinder captured pY and blockaded pY-mediated pathways in lung fibroblasts

**Figure 5:**
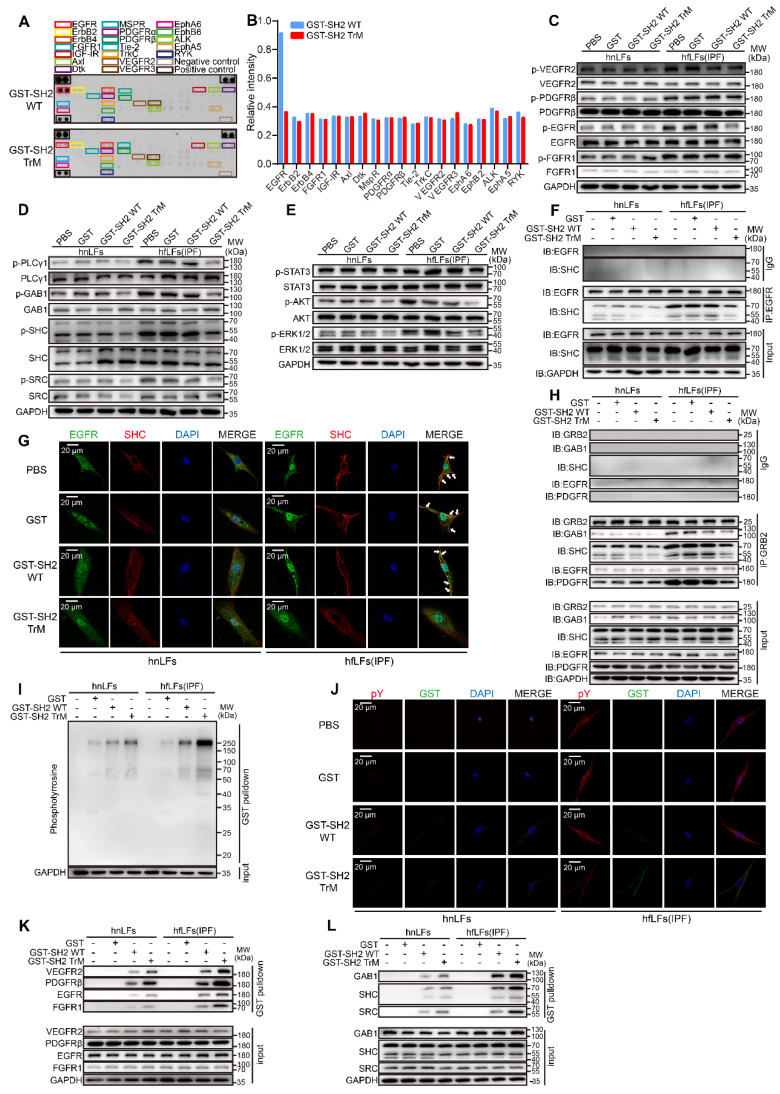
** SH2 superbinder blocked multiple fibrosis associated pathways through interrupting pY-SH2 combination in pY mediated signal transmission. A-B**, Phospho-RTK array analysis with 200 μg of lysates from hfLFs treated with 1 μM GST-SH2 WT or GST-SH2 TrM for 24 h. **C**, Western blot of p-VEGR2, p-PDGFRβ, p-EGFR and p-FGFR1 in hnLFs and hfLFs after GST, GST-SH2 WT and GST-SH2 TrM incubation. **D**, Western blot of p-PLCγ1, p-GAB1, p-SHC and p-SRC in hnLFs and hfLFs after GST, GST-SH2 WT or GST-SH2 TrM incubation. **E**, Western blot of p-AKT, p-ERK1/2 and p-STAT3 in hnLFs and hfLFs after GST, GST-SH2 WT or GST-SH2 TrM incubation. **F**, hnLFs and hfLFs were incubated with 1 μM GST, GST-SH2 WT or GST-SH2 TrM for 24 h. The immunoprecipitation of EGFR and SHC was detected. **G**, Representative fluorescence images of EGFR and SHC colocalization in hnLFs and hfLFs after GST, GST-SH2 WT or GST-SH2 TrM treatment. **H**, Immunoprecipitations of GRB2 with GAB1, SHC, EGFR and PDGFRβ. **I**, GST pull down assay showed the binding capacity of SH2 superbinder with pY in hnLFs and hfLFs. **J**, Representative fluorescence images of GST tag and pY colocalization in hnLFs and hfLFs after incubation with GST, GST-SH2 WT or GST-SH2 TrM. **K-L**, GST pull down assay showed the binding capacity of SH2 superbinder with RTKs (such as VEGFR2, PDGFRβ, EGFR and FGFR1) and adaptor proteins (such as GAB1, SHC and SRC) in hfLFs and hfLFs.

To clarify the mechanism of the remarkable inhibitory effect of SH2 superbinder, we next tried to explore the specific targets of SH2 superbinder. We confirmed that GST-SH2 TrM significantly decreased the pY levels in hfLfs and mfLfs whereas it had only slight effect on hnLfs or mnLfs (Figure 4F and Figure S5A). GST-SH2 TrM also reduced PHPS1-induced pY elevation in hfLfs (Figure S6A). Compared with GST-SH2 WT, GST-SH2 TrM did not suppress the pY levels of RTKs except for EGFR in hfLfs (Figure 5A-B). Western blot analysis further revealed that pY levels of VEGFR2 (Tyr1175), PDGFRβ (Tyr1021) and FGFR1 (Tyr654) were not suppressed by GST-SH2 TrM, but the pY levels of pEGFR (Tyr1068) were restrained in hfLfs and PHPS1-treated hnLfs (Figure 5C and Figure S6B). Meanwhile, in mfLfs, GST-SH2 TrM restrained pY levels of VEGFR2 (Tyr1175) and pEGFR (Tyr1068), rather than PDGFRβ (Tyr1021) or FGFR1 (Tyr654) (Figure S6C). These results suggested that the inhibition of GST-SH2 TrM on pY levels was not achieved by inhibiting autophosphorylation of RTKs. GST-SH2 TrM might act as an interrupter in RTK-adaptor complexes downstream. Next, we examined the pY levels of adaptor proteins and found that GST-SH2 TrM remarkably decreased their pY levels (Figure 5D), which were necessary for signal transduction of RTKs. This inhibition was dose- and time- dependent in hfLfs (Figure S6D-E). In addition, GST-SH2 TrM ultimately restrained common signaling pathways downstream of RTKs, included AKT, ERK1/2 and STAT3 pathways in hfLfs, and the suppression was dose- and time- dependent (Figure 5E and Figure S6F-G). In PHPS1 treated hnLfs, GST-SH2 TrM also inhibited the activation of these three pathways (Figure S6H). Moreover, the suppression of GST-SH2 TrM was also found in mfLfs (Figure S6I-J).

Next, we detected the protein-protein interactions mediated by pY-SH2 domain, which were essential for signal transmission. EGFR-SHC combination is decisive in the activation of EGFR/ERK signaling pathways 35. In hfLfs, there was a strong combination of EGFR with SHC, after treated with GST-SH2 TrM, the binding capacity was declined dramatically (Figure 5F). Quantitative analysis of immunofluorescence showed that only GST-SH2 TrM, not GST or GST-SH2 WT, restrained the co-localization of EGFR and SHC in hfLfs (Figure 5G and Figure S6K-L). Furthermore, GST-SH2 TrM suppressed combination of VEGFR2/GAB1, PDGFR/PLCγ1 and FGFR1/PLCγ1 both in hfLfs (Figure S6M-O) and PHPS1-stimulated hnLfs (Figure S6P-S). GRB2, another key adaptor protein, plays an essential role in pY-SH2 mediated RTKs signaling pathways 36. GST-SH2 TrM also blocked the binding of GRB2/EGFR, GRB2/PDGFR, GRB2/GAB1 and GRB2/SHC (Figure 5H). To ensure that various combinations were destroyed by GST-SH2 TrM rather than GST-SH2 WT via competitively binding to pY, we performed GST pull down assay. It was not surprising that GST-SH2 TrM could pull down more pY in hfLfs, which showed the powerful combination between GST-SH2 TrM and pY (Figure 5I). In PHPS1-stimulated hnLfs, GST-SH2 TrM also pulled down more pY, which inspired that only GST-SH2 TrM could specifically bind to pY up-regulated by PHPS1 (Figure S6T). Immunofluorescence demonstrated that GST-SH2 TrM could be combined with pY and decreased pY levels of hfLfs afterwards (Figure 5J and Figure S6-V). GST-SH2 TrM pulled down more RTKs including EGFR, VEGFR2, PDGFR and FGFR1 than GST or GST-SH2 WT (Figure 5K). In addition, GST-SH2 TrM also pulled down more adaptor proteins such as GAB1, SHC and SRC (Figure 5L). These data proved that GST-SH2 TrM could competitively bind to pY and interdict the abnormal combination of pY with SH2 domain, and further restrained multiple pY-related signaling pathways.

### SH2 superbinder exhibited excellent security by targeting lung fibroblasts based on pY levels *in vivo*

**Figure 6:**
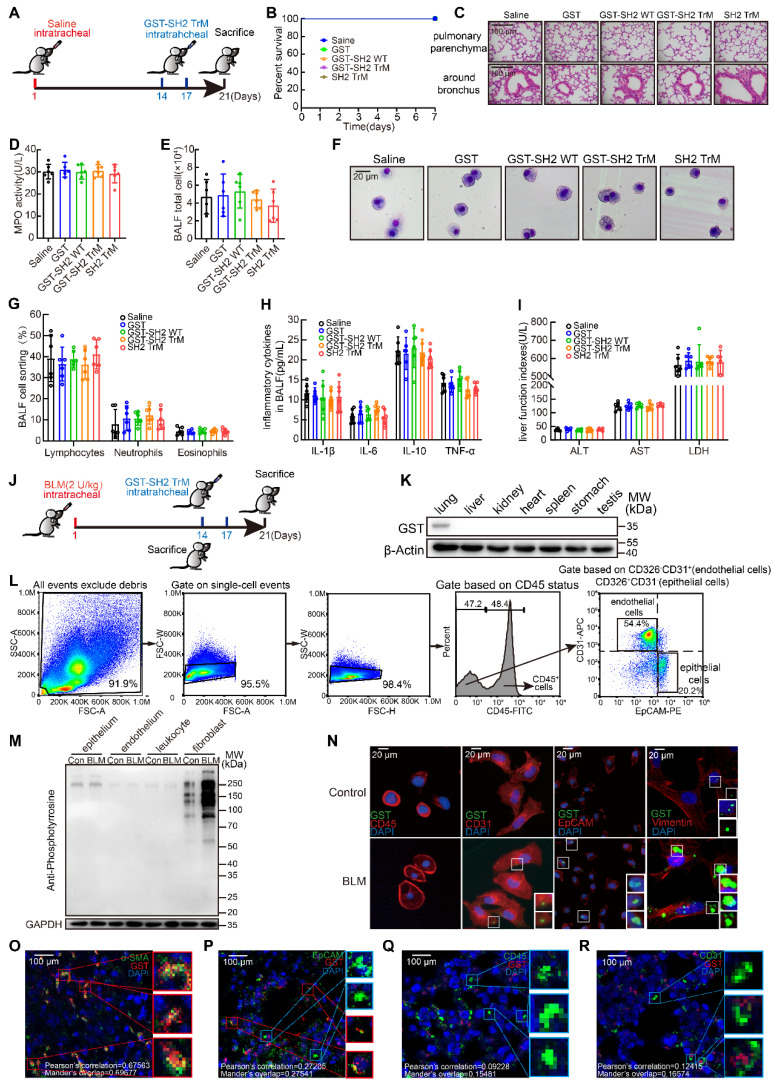
** SH2 superbinder exhibited excellent security by targeting lung fibroblasts based on pY levels *in vivo.* A**, Schematic showing progression of SH2 superbinder treatment in control mice. **B**, Percent survival during last 7 days after SH2 superbinder injection (n = 8). **C**, HE staining after SH2 superbinder challenge. Images showing the panoramic and partial view of lungs. **D**, Neutrophil accumulation measured by MPO assay in lung tissues after GST, GST-SH2 WT, GST-SH2 TrM or SH2-TrM treatment in saline groups (n = 6). **E**, Total cell counting in BALF (n = 6).** F-G**, Changes in different kinds of cells by Giemsa and Diff-quick staining in BALF (n = 6). **H**, Inflammatory cytokines in BALF measured by ELISA (n = 6). **I**, The determination of liver function index in serum (n = 6). **J**, Schematic showing progression of SH2 superbinder treatment in BLM-treated mice. **K**, Western blot of GST tag of different tissues in BLM group treated with GST-SH2 TrM. **L**, The process of FACS for mice lungs. **M**, Western blot of pY levels in different lung cells which were isolated by FACS of BLM treated 14 days mice. **N**, The fluorescence images of SH2 superbinder entering into activated fibroblasts, epithelial cells (EpCAM^+^), leukocytes (CD45^+^) and endothelial cells (CD31^+^). **O-R**, Representative images of SH2 superbinder in myofibroblast (α-SMA^+^) (**O**), epithelial cells (EpCAM^+^) (**P**), leukocytes (CD45^+^) (**Q**) and endothelial cells (CD31^+^) (**R**) of BLM-treated mice.

As a therapeutic drug, safety is the primary concern. We first detected the safety of GST-SH2 TrM. 8-week-old male C57BL/6 mice were intratracheally injected with saline. On day 14 and 17, we treated mice with intratracheal injection of GST, GST-SH2 WT, GST-SH2 TrM and SH2 TrM (5 mg/kg), and mice were sacrificed on day 21 (Figure 6A). GST tag is always used for better tracer and protein purification *in vitro*, but GST tag is about 26 KD and may disturb the effect of SH2 TrM *in vivo*. So, we also constructed SH2 TrM without GST tag digested by restriction enzyme for *in vivo* administration*.* All treatments did not change the survival of mice in saline groups (Figure 6B), and two GST-SH2 TrM intratracheal infusions did not cause inflammation or other abnormal phenotypes in trachea and lung parenchyma (Figure 6C). MPO activity of lung tissues (Figure 6D), total inflammatory cells counting and Giemsa stain of bronchoalveolar lavage fluid (BALF) also indicated that GST-SH2 TrM did not increase additional inflammatory cells (Figure 6E-G). Besides, inflammatory cytokines such as IL-1β, IL-6, IL-10 and TNF-α in BALF of GST-SH2 TrM group also keep unchanged compared with saline groups (Figure 6H). Through the measurements of alanine aminotransferase (ALT), aspartate transaminase (AST), lactate dehydrogenase (LDH) in the blood serum, we found that GST-SH2 TrM also did not induce liver damage in mice (Figure 6I). As a potential therapeutic agent, we should confirm the target cells of GST-SH2 TrM *in vivo*. We established a single dose BLM (2 U/kg) model for 21 days and treated mice with two intratracheal injections of GST-SH2 TrM (5 mg/kg) (Figure 6J). We barely detected GST-SH2 TrM in other organs except the lungs, which reflected that local intratracheal administration could avoid GST-SH2 TrM to diffuse into other organs and cause additional damage (Figure 6K). As SH2 superbinder could identify and combine with pY, we next detected pY levels of different cells in lung before GST-SH2 TrM injection. At day 14 after BLM intratracheal injection, we sacrificed mice and obtained epitheliums, endotheliums and leukocytes by flow cytometry and fibroblasts by tissue adherent culture (Figure 6L). Interestingly, pY levels only in fibroblasts from BLM treated mice were significantly increased than other cells (Figure 6M). Epitheliums, endotheliums, leukocytes and fibroblasts were then incubated with GST-SH2 TrM, and lots of GST-SH2 TrM could stay in fibroblasts, but few in epitheliums, endotheliums, leukocytes (Figure 6N). Meanwhile, more GST-SH2 TrM could combine in fibroblasts from BLM treated mice than control (Figure 6N). Through immunofluorescence of lung tissues, we re-confirmed that lots of GST-SH2 TrM targeted myofibroblasts, and few GST-SH2 TrM could enter into epitheliums, endotheliums and leukocytes (Figure 6O-R). All these data showed excellent myofibroblast targeting of GST-SH2 TrM in BLM model.

### SH2 superbinder attenuated multiple BLM-induced pulmonary fibrosis in mice

**Figure 7:**
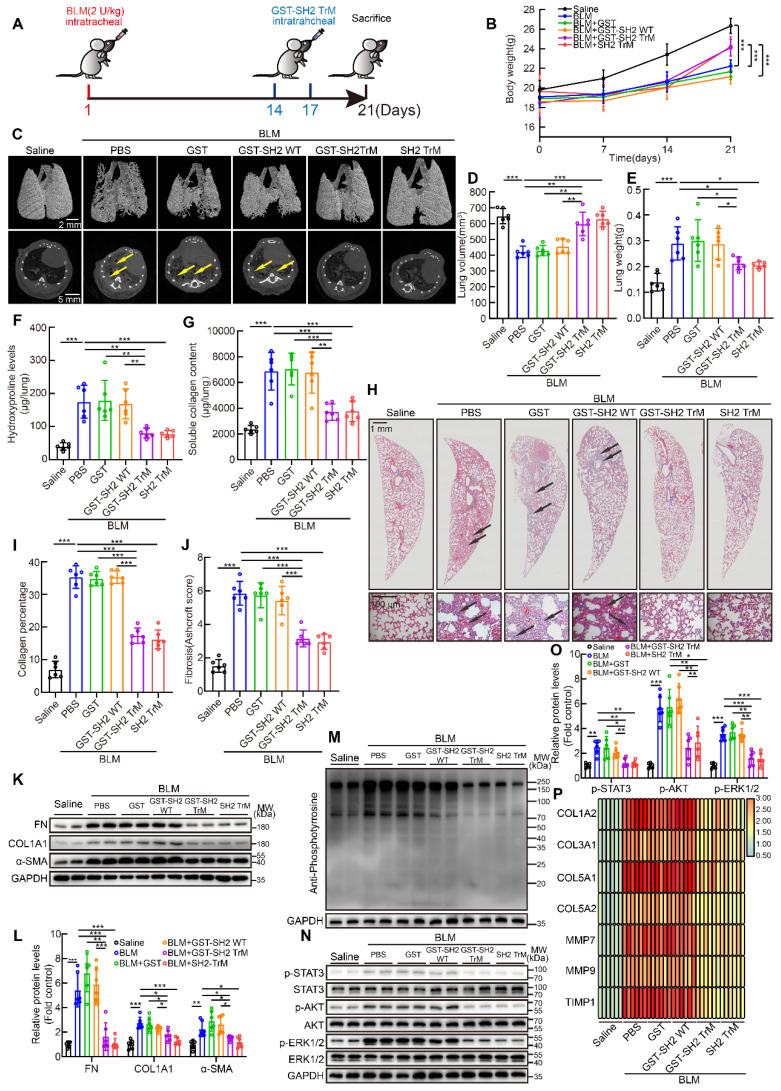
** SH2 superbinder restrained single dose intratracheal BLM-induced pulmonary fibrosis in mice.** After BLM (2 U/kg) treatment, PBS, GST, GST-SH2 WT, GST-SH2 TrM and SH2 TrM were intratracheally injected at day 14 and 17, all mice were sacrificed at day 21. The concentration of SH2 superbinder was 5 mg/kg, and injection volume was 50 μL per mouse. **A**, Schematic showing progression of SH2 superbinder treatment in single dose intratracheal BLM-induced pulmonary fibrosis. **B**, Body weights measurement every 7 days (n = 6). ^***^P < 0.001. **C**, Representative microCT reconstitution images of 3D and cross sections of lungs. Arrows showed the fibrosis foci which resulted in serious parenchymal destruction and volume reduction of lungs. **D**, Lung volume calculation by CTvox after 3D reconstitution of lungs (n = 6). ^**^P < 0.01, ^***^P < 0.001. **E**, Lung weights shown as bar charts (n = 6). ^*^P < 0.05, ^***^P < 0.01. **F**, Lung hydroxyproline content in different groups (n = 6).^ **^P < 0.01, ^***^P < 0.001. **G**, Soluble collagen content in different groups (n = 6). ^**^P < 0.01, ^***^P < 0.001. **H**, Masson's trichrome staining in different groups. Images showing the panoramic and partial view of lungs. Arrows pointed to the collagen deposition.** I-O**, Collagen percentage (**I**) and fibrosis score (**J**) of lungs after PBS, GST-SH2 WT, GST-SH2 TrM or SH2 TrM treatment (n = 6). ^***^P < 0.001. Western blot of FN, COL1A1 and α-SMA (**K-L**), pY (**M**), and p-STAT3, p-AKT and p-ERK1/2 (**N-O**) in lung tissues from different groups (n = 6). ^*^P < 0.05, ^**^P < 0.01, ^***^P < 0.001. **P**, Expression change of fibrosis associated genes after PBS, GST-SH2 WT, GST-SH2 TrM or SH2 TrM treatment (n = 6).

Next, we tried to evaluate the anti-fibrotic effect of SH2 superbinder in single dose BLM intratracheally injection model (Figure 7A). The body weight of mice in BLM group was significantly lower than that of control mice, but from day 14, when drugs were injected, body weight of mice treated with GST-SH2 TrM or SH2 TrM had a rapid increase than GST or GST-SH2 WT (Figure 7B). MicroCT scanning revealed that BLM injection resulted in lung destruction and volume reduction at day 21 (Figure 7C-D). Compared to GST or GST-SH2 WT administration, GST-SH2 TrM and SH2 TrM could preserve normal lung structure and attenuate the volume reduction induced by BLM. Likewise, the reduction in lung weight was observed (Figure 7E). Hydroxyproline content, soluble collagen content, Masson's trichrome staining and Ashcroft score showed that GST-SH2 TrM and SH2 TrM rather than GST or GST-SH2 WT markedly reduced the collagen deposition following BLM treatment (Figure 7F-J). Consistent with histological and biochemical data, intervention with GST-SH2 TrM and SH2 TrM at day 14 and 17 reduced the expression of FN, COL1A1 and α-SMA of lungs (Figure 7K-L). Furthermore, pY levels of lungs were also decreased upon GST-SH2 TrM or SH2 TrM administration (Figure 7M), as well as activation of STAT3, AKT and ERK1/2 (Figure 7N-O). The mRNA expressions of fibrosis associated genes, including *COL1A2*, *COL3A1*, *COL5A1*, *COL5A2*, *MMP7*, *MMP9* and *TIMP1*, were also suppressed by GST-SH2 TrM or SH2 TrM (Figure 7P). These data showed that both GST-SH2 TrM and SH2 TrM could resist lung fibrosis in 21 days acute BLM mouse model.

Single dose of BLM-induced pulmonary fibrosis could be reversed over time, which is distinctly different with irreversibility of IPF 23. To scrupulously confirm the effectiveness of GST-SH2 TrM, we established another repetitive intratracheal BLM model as previously reported 23. Mice were exposed to eight intratracheal doses of BLM (1 U/kg) to establish irreversible pulmonary fibrosis over 16 weeks. After final BLM injection, mice were treated with intratracheal injections of GST, GST-SH2 WT, GST-SH2 TrM or SH2 TrM twice a week for another two weeks (Figure S7A). GST-SH2 TrM or SH2 TrM administration at last two weeks could prevent the continuous declination of body weight in the multiple BLM stimulation mice (Figure S7B). MicroCT showed that long-term multiple BLM stimulation induced extremely serious lung destruction, meanwhile GST-SH2 TrM and SH2 TrM substantially mitigated the injury (Figure S7C). Besides, reduced lung volume and increased lung weight caused by BLM were also reversed by GST-SH2 TrM or SH2 TrM (Figure S7D-E). Both GST-SH2 TrM and SH2 TrM therapy reduced collagen deposition and parenchymal disruption by histological assessment and hydroxyproline assay, while no significant effect was found in GST or GST-SH2 WT group (Figure S7F-J). Furthermore, western blot of lung homogenates revealed that both GST-SH2 TrM and SH2 TrM reduced FN, COL1A1, α-SMA, pY, p-STAT3, p-AKT and p-ERK1/2 levels (Figure S7K-O). GST-SH2 TrM and SH2 TrM significantly down-regulated the mRNA levels of fibrosis associated genes (Figure S7P). Data above proved that GST-SH2 TrM and SH2 TrM markedly resisted long-term multiple BLM stimulation induced pulmonary fibrosis.

In addition to intratracheal BLM administration model, intraperitoneal BLM injection model was also employed. In this model, fibrosis foci appeared in subpleural area which corresponds with clinical characterization of IPF. Mice were intraperitoneally injected with BLM (40 U/kg) at day 1, 5, 8, 11 and 15 according to our previous study 24, and GST, GST-SH2 WT, GST-SH2 TrM or SH2 TrM were intratracheally administrated at day 24, 28, 34 and 36. At day 40, all mice were sacrificed (Figure S8A). The body weight of mice in BLM treated group was barely changed, but when GST-SH2 TrM or SH2 TrM applied, the weight of mice was increased from day 24 (Figure S8B). The results displayed that GST-SH2 TrM and SH2 TrM inhibited the formation of subpleural fibrosis (Figure S8C-J). In addition, the protein levels of fibrosis markers, pY, p-STAT3, p-AKT and p-ERK1/2 and mRNA of fibrosis associated genes, were also restrained by GST-SH2 TrM or SH2 TrM (Figure S8K-P). These results demonstrated that GST-SH2 TrM and SH2 TrM could reverse BLM intraperitoneal injection induced pulmonary fibrosis in mice.

In conclusion, SH2 superbinder effectively inhibited the development of BLM-induced pulmonary fibrosis in mice.

### SH2 superbinder exhibited better anti-fibrotic effect and fewer side-effects than nintedanib in mice

Nintedanib, as a multi-target RTKs (VEGFR, FGFR, PDGFR) inhibitor, has been approved by FDA for treatment for IPF. But nintedanib could only delay rather than stop the progression of IPF, and serious side-effects often limit the sustained medication. SH2 superbinder, as an inhibitor for pY-mediated RTK signaling pathways, had more targets and could enter into activated fibroblasts with high pY levels. So, we supposed that SH2 superbinder may have better therapeutic effect than nintedanib.

We compared the anti-fibrotic effect of nintedanib and SH2 superbinder *in vitro.* We found that these two drugs could suppress the proliferation and migration of hfLfs (Figure S9A-G). But GST-SH2 TrM could also restrain the differentiation of fibroblasts while nintedanib could not (Figure S9H-L). Nintedanib and GST-SH2 TrM also reduced protein levels of FN and COL1A1, but nintedanib could not reduce protein level of α-SMA (Figure S9M). Whole pY levels were slightly down-regulated by nintedanib, while GST-SH2 TrM significantly inhibited pY levels (Figure S9N). Although GST-SH2 TrM did not restrain phosphorylation levels of RTKs, it had wider repression for downstream signaling pathways associated with fibrosis (Figure S9O-P).

**Figure 8:**
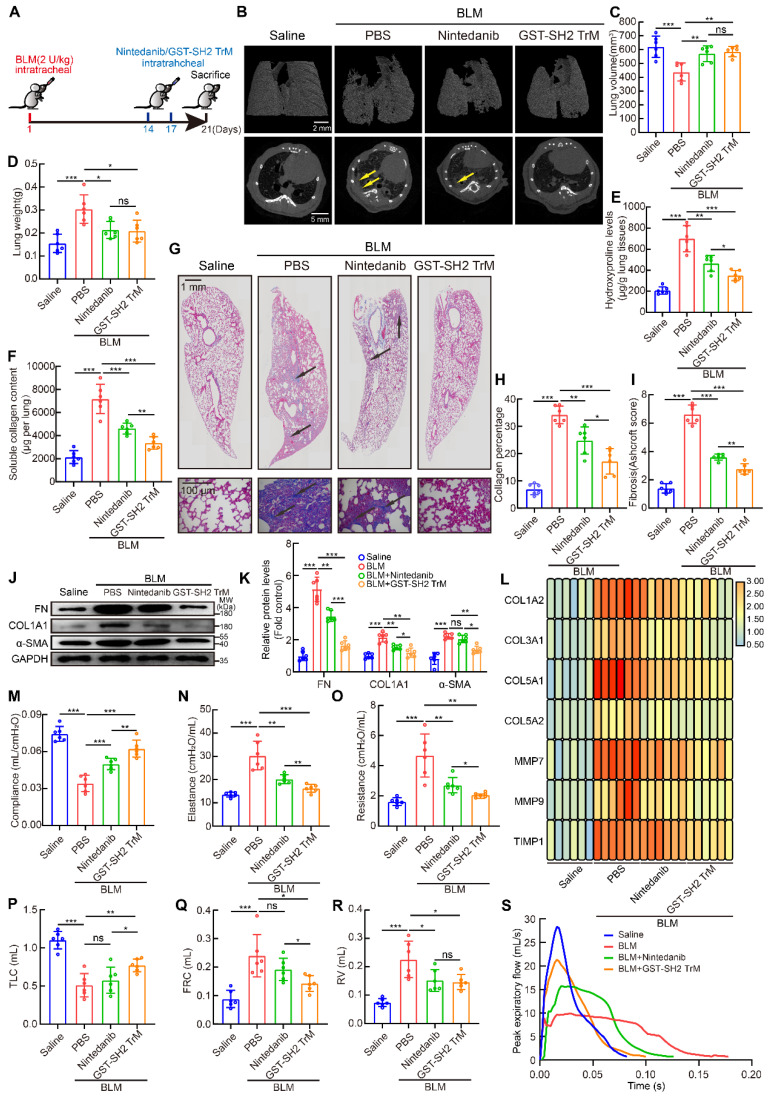
** SH2 superbinder had better anti-fibrotic effect than nintedanib.** After BLM (2 U/kg) treatment, PBS, GST-SH2 TrM (5 mg/kg) and nintedanib (20 mg/kg) were intratracheally injected at day 14 and 17, all mice were sacrificed at day 21. **A**, Schematic showing progression of GST-SH2 TrM and nintedanib in this model. **B**, Representative microCT reconstitution images of 3D and cross sections of lungs. Arrows showed the fibrosis foci which resulted in serious parenchymal destruction and volume reduction of lungs. **C**, Lung volume calculation by CTvox after 3D reconstitution of lungs (n = 6). ^**^P < 0.01, ^***^P < 0.001. **D**, Lung weights shown as bar charts (n = 6). ^*^P < 0.05, ^***^P < 0.001. **E**, Lung hydroxyproline content in different groups (n = 6). ^*^P < 0.05, ^**^P < 0.01, ^***^P < 0.001. **F**, Soluble collagen content in different groups (n = 6). ^**^P < 0.01, ^***^P < 0.001. **G**, Masson's trichrome staining in different groups. Images showing the panoramic and partial view of mice lung. Arrows pointed to the collagen deposition. **H-I**, Collagen percentage (**H**) and fibrosis score (**I**) of lungs after PBS, GST-SH2 WT, GST-SH2 TrM or SH2 TrM treatment (n = 6). ^*^P < 0.05, ^**^P < 0.01, ^***^P < 0.001. **J-K**, Western blot of FN, COL1A1 and α-SMA in lung tissues from different groups (n = 6). ^*^P < 0.05, ^**^P < 0.01, ^***^P < 0.001. **L**, Expression change of fibrosis associated genes after PBS, nintedanib or GST-SH2 TrM treatment (n = 6). **M-O**, Lung compliance (**M**), elastance (**N**) and resistance (**O**) were measured using Forced Manoeuvres System (n = 6). ^*^P < 0.05, ^**^P < 0.01, ^***^P < 0.001. **P-S**, TLC (**P**), FRC (**Q**), RV (**R**) and PEF (**S**) were compared between nintedanib or GST-SH2 TrM treated mice (n = 6). ^*^P < 0.05, ^**^P < 0.01, ^***^P < 0.001.

Then we used single dose BLM (2 U/kg) model to contrast the anti-fibrotic effects of the two drugs *in vivo* (Figure 8A). From microCT scan, we found that GST-SH2 TrM had better protection against lung destruction than nintedanib (Figure 8B). But no difference was found in lung volume and weight between GST-SH2 TrM and nintedanib treatments (Figure 8C-D). Hydroxyproline content, soluble collagen content, Masson's trichrome staining and Ashcroft score showed that GST-SH2 TrM had better therapeutic effects than nintedanib in BLM-treated mice (Figure 8E-I). Fibrosis markers detection also revealed that GST-SH2 TrM had more effective inhibition than nintedanib (Figure 8J-L). Pulmonary fibrosis eventually leads to impaired lung function. We next used EMMS Forced Manoeuvres System to compare the effect of two drugs on improving lung function. Although GST-SH2 TrM and nintedanib could improve lung compliance, reduce lung elastance and resistance, GST-SH2 TrM displayed more outstanding effect (Figure 8M-O). Besides, GST-SH2 TrM also exhibited better efficient than nintedanib in recovery of TLC and FRC (Figure 8P-Q). There was no difference in the ability to reduce RV in the two groups (Figure 8R), while GST-SH2 TrM treated mice showed better expiratory function (Figure 8S).

In addition to therapeutic effect, side-effects also should be paid attention. In BLM-treated groups, GST-SH2 TrM was better at preventing weight loss of mice (Figure S10A). Nintedanib could result in impairment of liver function, but GST-SH2 TrM remained better liver function after two administrations (Figure S10B-D). Besides, GST-SH2 TrM also had an edge over nintedanib in the prevention of decreased white blood cell count and intestinal villi damage (Figure S10E-F).

All these data proved that GST-SH2 TrM was efficient and safer than nintedanib in the treatment of pulmonary fibrosis in mice.

## Discussion

In this study, our bioinformatic analysis revealed that the cytokines and growth factors signaling pathways were strongly associated with IPF. By detecting cytokines signaling activation induced AA phosphorylation, high pY levels rather than pS/pT were found in lung fibroblasts from IPF patients and BLM-treated mice. PY-induced multiple signaling pathways were activated through the binding of SH2 domain. SH2 superbinder (GST-SH2 TrM) with a strong binding capacity to pY than wild type SH2 domain, blocked the downstream signaling pathways of AKT, ERK1/2 and STAT3, and ultimately suppressed the proliferation, migration, differentiation of lung fibroblasts as well as ECM deposition (Figure S10G).* In vivo*, SH2 superbinder showed an excellent anti-fibrotic effect in multiple BLM-challenged models and a better therapeutic efficacy than nintedanib with fewer side-effects.

Lung fibroblasts which exist in ECM-rich interstitial spaces are the key effectors of pulmonary fibrosis 37, 38. They are abnormally activated in proliferation, migration, apoptosis and differentiation under a profibrotic microenvironment 39-41. In addition to TGF-β, other cytokines and growth factors have been proved involving in activation of fibroblasts in recent years. Benjamin *et al*. confirmed that IL-11-stimulated lung fibroblasts became invasive phenotype through ERK signaling pathway in pulmonary fibrosis 42. IL-13, as a potential activator of fibroblasts, induces ECM over-synthesis 43. Nonetheless, growth factor pathways are also involved in pathogenesis and progress of pulmonary fibrosis. The activation of EGFR signaling leads to lung fibrosis after SARS coronavirus infection 44. PDGF has an essential effect on the expansion of myofibroblasts through promoting cell proliferation and migration 45. Fibroblast growth factor-2 (FGF-2) is a potent mitogen and induces collagen synthesis in lung fibroblasts and myofibroblasts 46, 47. Blocking VEGF, PDGF and FGF signaling pathways displays anti-fibrotic effect by suppressing fibroblasts proliferation 38, 47, 48. These studies evidently suggest that the effects of cytokines and growth factors on lung fibroblasts are direct inducements of pulmonary fibrosis.

Growth factors signal transduction is often initiated by RTKs, which plays a critical role in activating intracellular signaling cascades 49. The signal transduction triggered by majority cytokines is mediated by JAK/STAT signaling pathway 50. SH2 domain is essential in both RTKs and JAK/STAT signaling pathways. Through binding to pY, SH2 domain establishes connections between different components and then phosphorylation is transmitted sequentially 13. SH2 superbinder with triple AA mutants was designed to target this specific binding process 14. *In vitro* and *in vivo* experiments showed that SH2 superbinder had stronger affinity than natural SH2 domain to pY 14, 51. In another study, we found that GST-SH2 TrM also restrained TGF-β1-induced differentiation of fibroblasts, despite that the receptor of TGF-β is serine/threonine kinase and the signal transduction is dependent on pS/pT. Although the inhibition mechanism of GST-SH2 TrM in pS/pT-mediated TGF-β pathway is not clear, recent study has indicated that there is a complex crosstalk between pY-mediated and pS/pT-mediated signal transmissions 52. We also tried to further uncover the deeper mechanism in our study. Furthermore, GST-SH2 TrM had little effect on normal lung fibroblasts. When GST-SH2 TrM entered normal lung fibroblasts, it did not accumulate. Under low pY levels environment, excessive GST-SH2 TrM that does not bind to pY will egress the cells through cell membranes. In another word, GST-SH2 TrM did not accumulate in normal lung fibroblasts. In our study, we also found that SH2 superbinder could decrease pY levels of EGFR rather than other RTKs. According to the binding pattern of SH2 superbinder with pY, SH2 superbinder could recognize pY, block combination of pY and WT SH2 domain and ultimately suppress signal transmission. This pattern of suppression is different from small molecule inhibitors that bind to ATP-binding sites of RTKs and restrain autophosphorylation of tyrosine residues. SH2 superbinder inhibits pY levels of downstream proteins rather than its binding proteins. Our results showed significant inhibitory effect of SH2 superbinder on pY levels of RTKs downstream proteins such as GAB1, GRB2 and SHC. In lung fibroblasts cells, SH2 superbinder could specifically restrain pY levels of EGFR, while in our previous study on pancreatic ductal adenocarcinoma cells, SH2 superbinder suppressed pY levels of VEGFR, EGFR and IGFR 51. We think that there is a feedback regulation mechanism between pY levels of RTKs and SH2 superbinder in different cell types and future study should be done to clarify the mechanism.

Nintedanib and pirfenidone, the only two FDA-approved drugs for IPF, also target cytokines and growth factors related pathways 48, 53. As a multi-target RTKs inhibitor, nintedanib, slows down the progression of pulmonary fibrosis. Unlike the direct inhibition of RTKs activity by nintedanib, SH2 superbinder restrains pY-mediated signal transmission by blocking the binding of pY with SH2 domain, resulting in a stronger obstruction to pY-mediated signaling than nintedanib. PY had been proved to participate in fibrosis in recent studies. SHP-2, as a protein tyrosine phosphatase, catalyzes the dephosphorylation of tyrosine. Reduction of SHP-2 activity or expression will promote differentiation of lung fibroblasts 34. Loss of SHP-2 in alveoli epithelia or macrophages also results in pulmonary fibrosis in mice 54, 55. These studies suggested that when the balance of tyrosine phosphorylation was broken, increased pY levels could induce fibrosis, which is consistent with our findings. Therefore, characterizing pY-mediated signaling pathways deserves more attention for revealing the pathogenesis of IPF and development of new drugs.

In recent years, more protein and peptide drugs have been used in clinical disease treatment. Compared with small molecule drugs, protein and peptide drugs display higher bioactivity and specificity and lower toxicity 56. Protein and peptide drugs such as neutralizing anti-IL-11 antibody and caveolin-1-derived peptide exhibit excellent anti-fibrotic effect in mouse model of pulmonary fibrosis 6, 42. However, wide application of protein and peptide drugs in clinic is constrained because of the unstable physicochemical properties of proteins. They are susceptible to the change of PH value, enzymatic degradation and rapid elimination from circulation 56. As a protein prodrug, SH2 superbinder was proved to be safer than nintedanib *in vivo*. We treated mice with GST-SH2 TrM at 1, 5 and 10 mg/kg. The dose of 1 mg/kg did not significantly inhibit lung fibrosis whereas 10 mg/kg made the mice listless and dull. The 5 mg/kg dose of intratracheal injection of GST-SH2 TrM was suitable for anti-fibrosis without damaging in healthy cells. In addition, the timing of administration is also important. In this work, we discovered that fibroblasts expressed high pY levels only at day 14, which provided specific cell targeting for SH2 superbinder. Therefore, we administered two injections of GST-SH2 TrM at day 14 and day 17 after the mice being BLM treated. Since pY levels of various cells at different stages in BLM model are different, choosing the right time for medications is important to ensure efficacy and prevent side-effects.

In our previous report, protein degradation was the main challenge. In this study, we performed multiple intratracheal administrations to avoid SH2 superbinder degradation. However, better ways to prevent degradation should be developed in clinical settings. Further studies are recommended to focus on elongating the half-life of SH2 superbinder. Hettiarachchi and colleagues found a specific ligand of fibroblast activation protein (FAPL) could act as a delivery for myofibroblast in IPF 57. Inspired by this work, we are also trying to further increase the target of SH2 superbinder. Because of the different inhibitory mechanisms of small molecule inhibitors, combining SH2 superbinder with nintedanib or pirfenidone may further reduce the dose and frequency requirements and increase the sensitivity and improve therapeutic effectiveness of the drug. Hence, seeking better drug delivery and combination mode is the core of our future research.

In conclusion, SH2 superbinder, as an inhibitor for multiple pY-mediated signaling pathways, has significant anti-fibrotic effect of lung. We believe that SH2 superbinder will be a promising prodrug, which can be applied in treating patients with IPF in the future.

## Supplementary Material

Supplementary figures and table.

## Abbreviations

IPF: idiopathic pulmonary fibrosis
FDA: food and drug administration
pY: phosphotyrosine
pS: phosphoserine
pT: phosphothreonine
SH2: src homology-2
AA: amino acid
ECM: extracellular matrix
TGF-β: transforming growth factor-β
TK: tyrosine kinase
RTK: receptor tyrosine kinase
NRTK: non-receptor tyrosine kinase
PTB: phosphotyrosine-binding
CPP: cell penetrating peptide
CCK-8: cell counting kit-8
TEM: transmission electron microscopy
BLM: bleomycin
FRC: functional residual capacity
IC: inspiratory volume
RV: residual volume
TLC: total lung capacity
MPO: myeloperoxidase
ALT: alanine aminotransferase
AST: aspartate aminotransferase
LDH: lactic dehydrogenase
