# Supplementary material for: Blockade of phosphotyrosine pathways suggesting SH2 superbinder as a novel therapy for pulmonary fibrosis
Source: Theranostics. 2022 May 26;12(10):4513–35. doi: 10.7150/thno.72269 (PMC9254236; doi:10.7150/thno.72269)
Supplement: Supplementary file 1 — Supplementary figures and table. [file thnov12p4513s1.pdf]

Supplementary material for

Blockade of phosphotyrosine pathways suggesting SH2 superbinder as a novel therapy for pulmonary fibrosis

Meng Wang[1, #], An-Dong Liu[2, #], Qian Niu[3], Xiao Feng[1], Yuan-Yi Zheng[1], Shuai-Jun Chen[1], Hui Xu[4], Qian Li[1], Guo-Qing Hou[2], Xiao-Yang Bi[2], Yu-Zhi Lu[3], Pei-Pei Cheng[1], Li-Mei Liang[3], Ye-Han Jiang[3], Li-Qin Zhao[3], Fei Liu[1], Lin-Jie Song[3], Li-Ling Zhou[3], Ling-Yan Xiao[5], Feng Chen[6], Shawn Shun-Cheng Li[7], Wan-Li Ma[3, 8, *] Xuan Cao[2, **], Hong Ye[1, 8, ***].

[*]Corresponding author. Email: whmawl@ hust.edu.cn (Wan-Li Ma); caoxuan@hust.edu.cn (Xuan Cao); yehmwl@hust.edu.cn (Hong Ye)

Figure S1. Cytokine and growth factor signaling pathways were included in IPF.

Figure S2. High pY levels promoted the proliferation, migration and differentiation of lung fibroblasts from mice.

Figure S3. Concentration and time dependent effects of SH2 superbinder for inhibition of proliferation, migration and differentiation in lung fibroblasts.

Figure S4. SH2 superbinder could restrain proliferation, migration and differentiation of lung fibroblasts induced by PHPS1.

Figure S5. SH2 superbinder suppressed proliferation, migration and differentiation of lung fibroblasts from mice.

Figure S6. SH2 superbinder could inhibit combination of RTKs with adaptor proteins and signaling pathways downstream, which depends on pY-SH2 binding.

Figure S7. SH2 superbinder limited repetitively intratracheal BLM-induced pulmonary fibrosis in mice.

Figure S8. SH2 superbinder reversed intraperitoneal BLM-induced pulmonary fibrosis in mice.

Figure S9. SH2 superbinder had effective inhibition of proliferation, migration and differentiation than nintedanib.

Figure S10. SH2 superbinder had fewer side-effects than nintedanib *in vivo*.

Table S1. Patient demographics

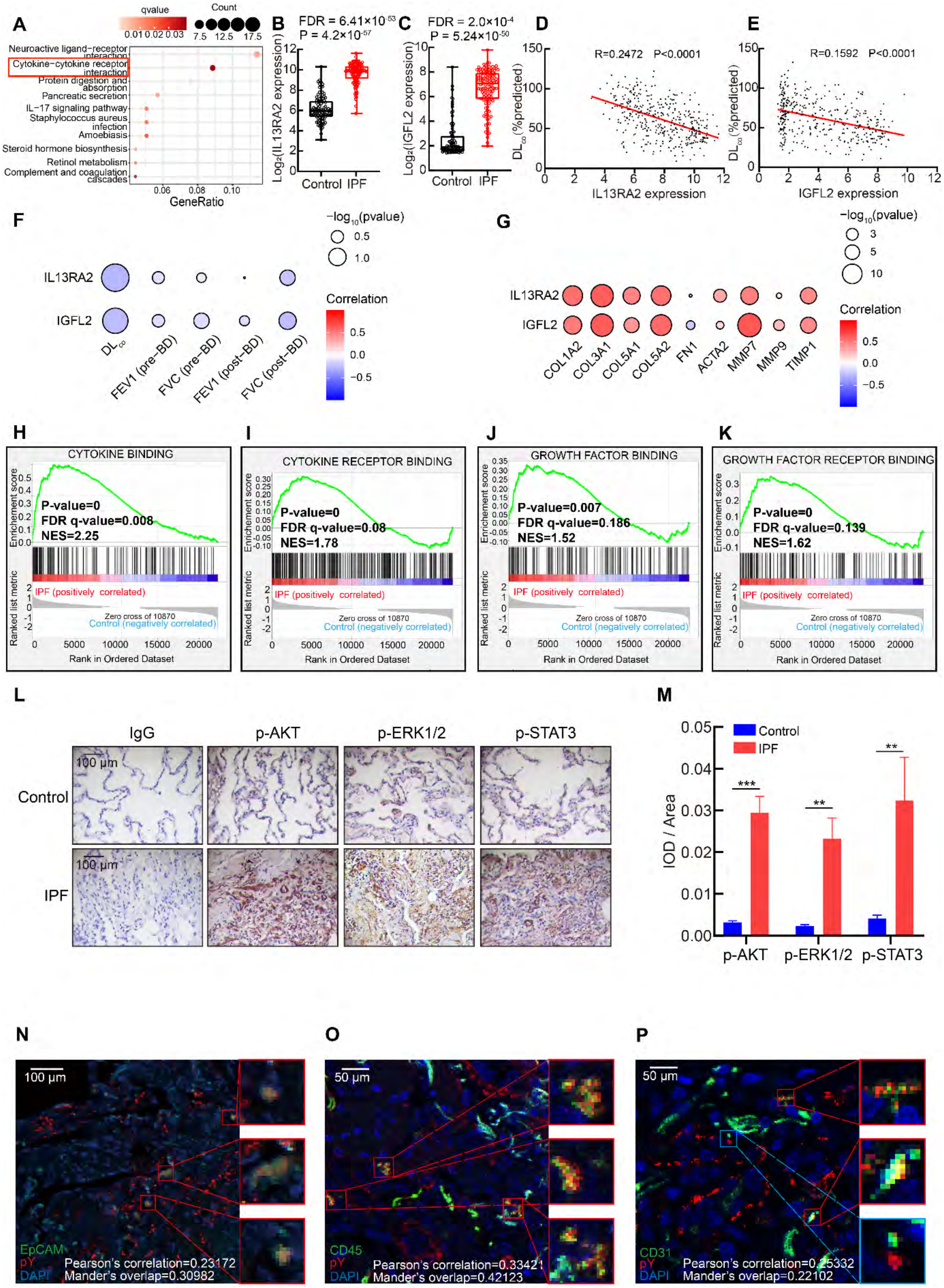
A
qvalue
0.01 0.02 0.03
Count
7.5 12.5 17.5
Neuroactive ligand−receptor interaction
Cytokine−cytokine receptor interaction
Protein digestion and absorption
Pancreatic secretion
IL−17 signaling pathway
Staphylococcus aureus infection
Amoebiasis
Steroid hormone biosynthesis
Retinol metabolism
Complement and coagulation cascades
0.06 0.08 0.10
GeneRatio
B
FDR = 6.41×10-53
P = 4.2×10-57
Log2(IL13RA2 expression)
Control IPF
C
FDR = 2.0×10-4
P = 5.24×10-50
Log2(IGFL2 expression)
Control IPF
D
R=0.2472 P<0.0001
DLco (%predicted)
IL13RA2 expression
E
R=0.1592 P<0.0001
DLco (%predicted)
IGFL2 expression
F
−log10(pvalue)
0.5
1.0
IL13RA2
IGFL2
DLco
FEV1 (pre-BD)
FVC (pre-BD)
FEV1 (post-BD)
FVC (post-BD)
Correlation
0.5
0.0
−0.5
G
−log10(pvalue)
3
5
10
IL13RA2
IGFL2
COL1A2
COL3A1
COL5A1
COL5A2
FN1
ACTA2
MMP7
MMP9
TIMP1
Correlation
0.5
0.0
−0.5
H
CYTOKINE BINDING
P-value=0
FDR q-value=0.008
NES=2.25
I
CYTOKINE RECEPTOR BINDING
P-value=0
FDR q-value=0.08
NES=1.78
J
GROWTH FACTOR BINDING
P-value=0.007
FDR q-value=0.186
NES=1.52
K
GROWTH FACTOR RECEPTOR BINDING
P-value=0
FDR q-value=0.139
NES=1.62
Enrichment score
Ranked list metric
IPF (positively correlated)
Zero cross of 10870
Control (negatively correlated)
Rank in Ordered Dataset
L
IgG
p-AKT
p-ERK1/2
p-STAT3
Control
IPF
100 μm
M
Control
IPF
IOD / Area
***
**
**
p-AKT
p-ERK1/2
p-STAT3
N
100 μm
EpCAM
pY
DAPI
Pearson's correlation=0.23172
Mander's overlap=0.30982
O
50 μm
CD45
pY
DAPI
Pearson's correlation=0.33421
Mander's overlap=0.42123
P
50 μm
CD31
pY
DAPI
Pearson's correlation=0.25332
Mander's overlap=0.22102

Figure S1. Cytokine and growth factor signaling pathways were included in IPF. A, KEGG pathway enrichment analysis with lung function (DL_{co}%) of IPF patients in GSE47460. **B-C,** Log-transformed normalized gene expression values of *IL13RA2* and *IGFL2* in lung tissues from patients with IPF and control in GSE47460. **D-E,** Linear relationship and association between expressions of *IL13RA* and *IGFL2* and lung functions (DL_{co}%). **F,** Association between expressions of *IL13RA* and *IGFL2* and other pulmonary function indicators as FEV1 pre-BD, FVC pre-BD, FEV1 post-BD, FVC post-BD. FEV1, forced expiratory volume; BD, bronchodilation; FVC, forced vital capacity. **G,** Association between expressions of *IL13RA* and *IGFL2* and other fibrosis related genes. **H-K,** GSEA analysis of lung fibroblasts from IPF patients in dataset GSE71351. **L-M,** Representative images of p-AKT, p-ERK1/2 and p-STAT3 immunostaining of lung tissue sections from IPF patients and healthy control (n = 3). $^{**}P < 0.01$, $^{***}P < 0.001$. IgG was an isotype control for p-AKT, p-ERK1/2 and p-STAT3. **N-P,** Representative images of pY in epithelial cells ($EpCAM^+$) (**N**), leukocytes ($CD45^+$) (**O**) and endothelial cells ($CD31^+$) (**P**) of IPF patients.

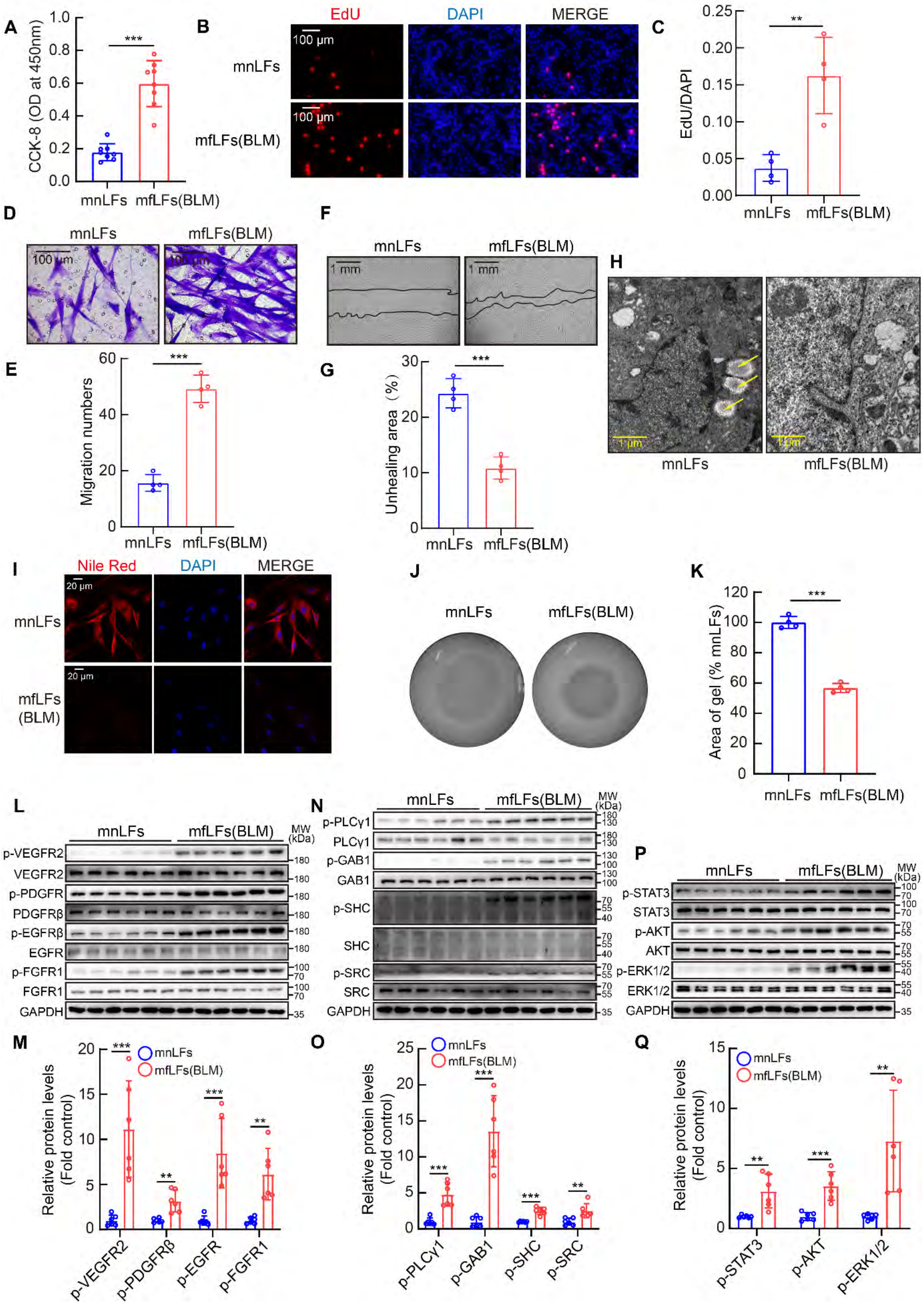
A
CCK-8 (OD at 450nm)
mnLFs
mfLFs(BLM)
B
EdU
DAPI
MERGE
100 μm
C
EdU/DAPI
D
E
Migration numbers
F
1 mm
G
Unhealing area (%)
H
1 μm
I
Nile Red
20 μm
J
K
Area of gel (% mnLFs)
L
p-VEGFR2
VEGFR2
p-PDGFR
PDGFRβ
p-EGFRβ
EGFR
p-FGFR1
FGFR1
GAPDH
MW (kDa)
M
Relative protein levels (Fold control)
N
p-PLCγ1
PLCγ1
p-GAB1
GAB1
p-SHC
SHC
p-SRC
SRC
O
P
p-STAT3
STAT3
p-AKT
AKT
p-ERK1/2
ERK1/2
Q

Figure S2. High pY levels promoted the proliferation, migration and differentiation of lung fibroblasts from mice. A-C, Changes in cell proliferation between fibroblasts between mnLFs and mfLFs by CCK-8 and EdU assay (n = 7 or 4). $^{**}P < 0.01$, $^{***}P < 0.001$. **D-G,** Comparison of migration between mnLFs and mfLFs (n = 4). $^{***}P < 0.001$. **H-I,** Differentiation measured by content of lipid droplets through TEM and Nile red staining between mnLFs and mfLFs. **J-K,** The contractility of mnLFs and mfLFs in 3D collagen matrices (n = 4). $^{***}P < 0.001$. **L-M,** Western blot and quantification of p-VEGR2, p-PDGFRβ, p-EGFR and p-FGFR1 in mnLFs and mfLFs (n = 4). $^{**}P < 0.01$, $^{***}P < 0.001$. **N-O,** Western blot and quantification of p-PLCγ1, p-GAB1, p-SHC and p-SRC in mnLFs and mfLFs (n = 6). $^{**}P < 0.01$, $^{***}P < 0.001$. **P-Q,** Western blot and quantification of p-AKT, p-ERK1/2 and p-STAT3 in mnLFs and mfLFs (n = 6). $^{**}P < 0.01$, $^{***}P < 0.001$.

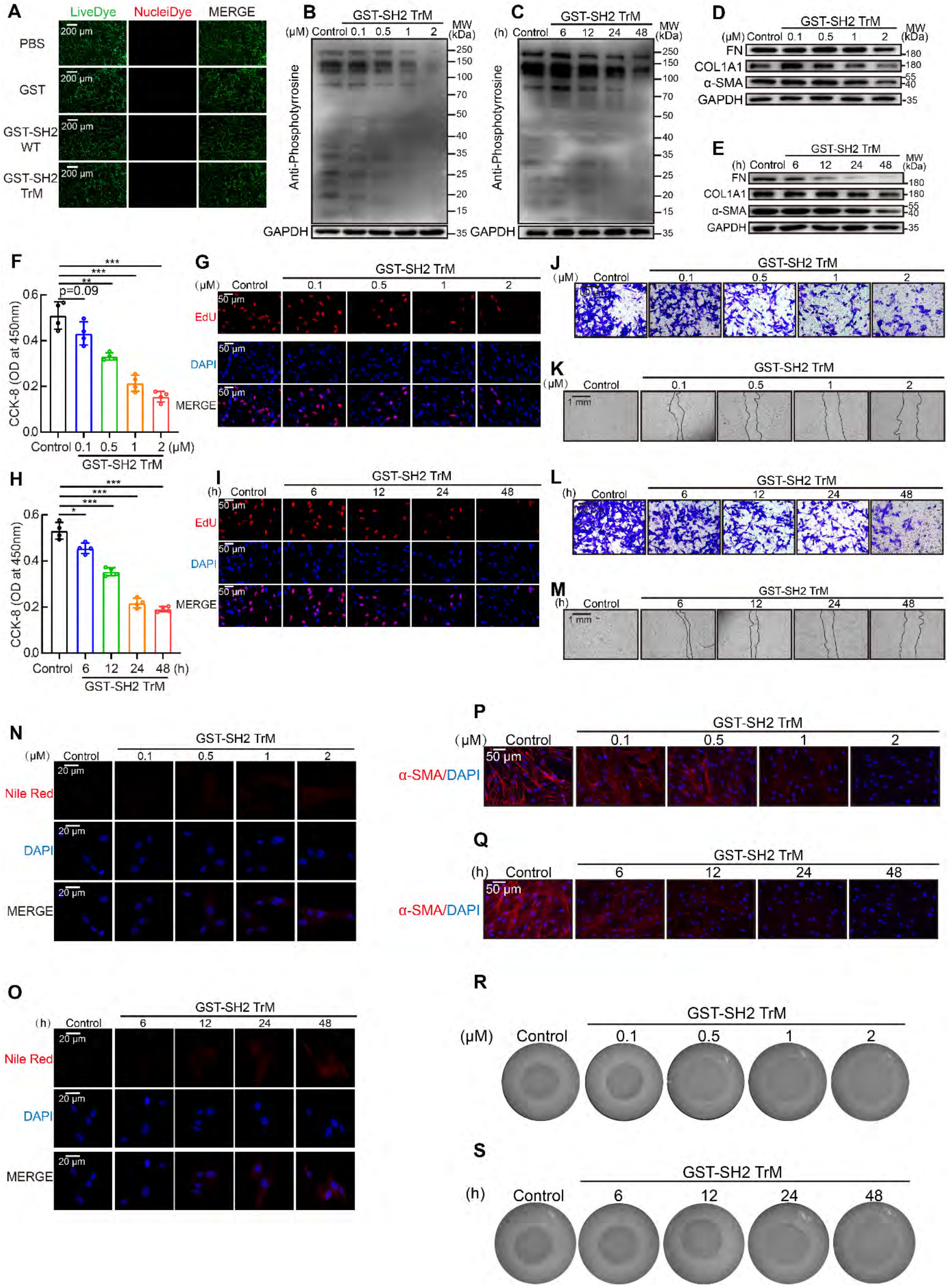
A
LiveDye
NucleiDye
MERGE
PBS
GST
GST-SH2 WT
GST-SH2 TrM
200 μm
B
GST-SH2 TrM
(μM) Control 0.1 0.5 1 2
MW (kDa)
Anti-Phosphotyrrosine
GAPDH
C
GST-SH2 TrM
(h) Control 6 12 24 48
D
(μM) Control 0.1 0.5 1 2
FN
COL1A1
α-SMA
GAPDH
E
(h) Control 6 12 24 48
F
CCK-8 (OD at 450nm)
p=0.09
Control 0.1 0.5 1 2 (μM)
GST-SH2 TrM
G
EdU
DAPI
MERGE
50 μm
H
Control 6 12 24 48 (h)
I
J
K
1 mm
L
M
N
Nile Red
20 μm
O
P
α-SMA/DAPI
Q
R
S

Figure S3. Concentration and time dependent effects of SH2 superbinder for inhibition of proliferation, migration and differentiation in lung fibroblasts. A, Cytotoxic effects of GST, GST-SH2 WT or GST-SH2 TrM in hnLFs. Live cells were stained green, and dead cells were stained red. **B-C,** Concentration and time dependent effects of GST-SH2 TrM on pY levels in hfLFs. **D-E,** Concentration and time dependent effects of GST-SH2 TrM on FN, COL1A1 and α-SMA protein levels in hfLFs. **F-I,** Concentration and time dependent effects of GST-SH2 TrM on proliferation of hfLFs. **J-M,** Concentration and time dependent effects of GST-SH2 TrM on migration of hfLFs. **N-Q,** Concentration and time dependent effects of GST-SH2 TrM on differentiation of hfLFs. **R-S,** Concentration and time dependent effects of GST-SH2 TrM on contractility of hfLFs.

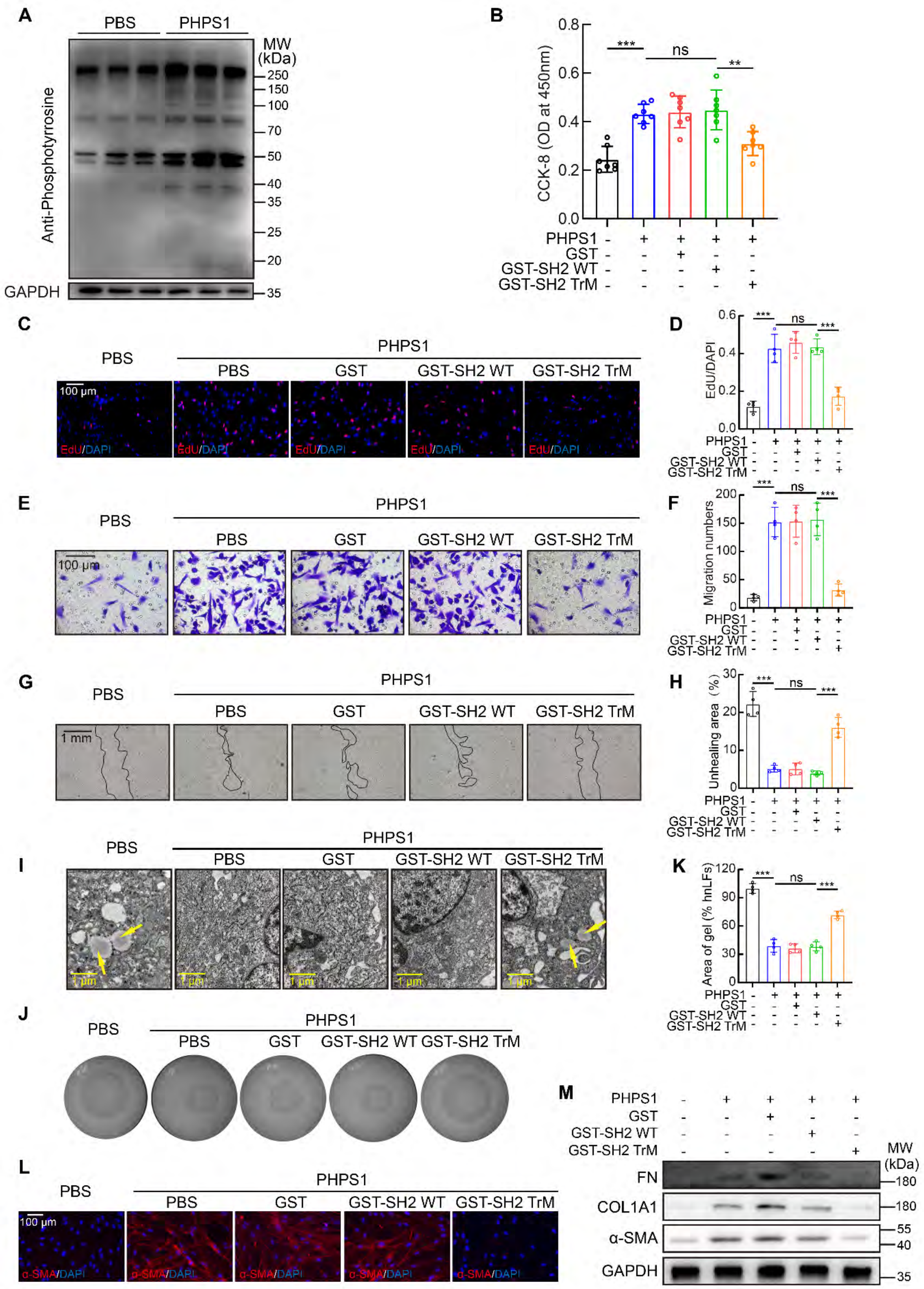

A
PBS
PHPS1
MW (kDa)
250
150
100
70
50
40
35
25
20
Anti-Phosphotyrosine
GAPDH
35
B
CCK-8 (OD at 450nm)
***
ns
**
PHPS1
GST
GST-SH2 WT
GST-SH2 TrM
C
PBS
PHPS1
PBS
GST
GST-SH2 WT
GST-SH2 TrM
100 μm
EdU/DAPI
D
EdU/DAPI
E
100 μm
F
Migration numbers
G
1 mm
H
Unhealing area (%)
I
1 μm
K
Area of gel (% hnLFs)
J
L
100 μm
α-SMA/DAPI
M
FN
COL1A1
α-SMA
GAPDH
180
55
40

Figure S4. SH2 superbinder could restrain proliferation, migration and differentiation of lung fibroblasts induced by PHPS1. Pretreatment of hnLFs with PHPS1 10 μM for 24 h and then GST, GST-SH2 WT or GST-SH2 TrM were incubated for another 24 h. **A,** Western blot showed the change of pY levels in PHPS1 treated hnLFs. **B-D,** The effect of GST-SH2 TrM on proliferation of PHPS1 treated hnLFs (n = 7 or 4). $^{**}P < 0.01$, $^{***}P < 0.001$. **E-H,** The effect of GST-SH2 TrM on migration of PHPS1 treated hnLFs (n = 4). $^{***}P < 0.001$. **I-L,** The effect of GST-SH2 TrM on differentiation of PHPS1 treated hnLFs (n = 4). $^{***}P < 0.001$. **M,** The effect of GST-SH2 TrM on ECM production of PHPS1 treated hnLFs.

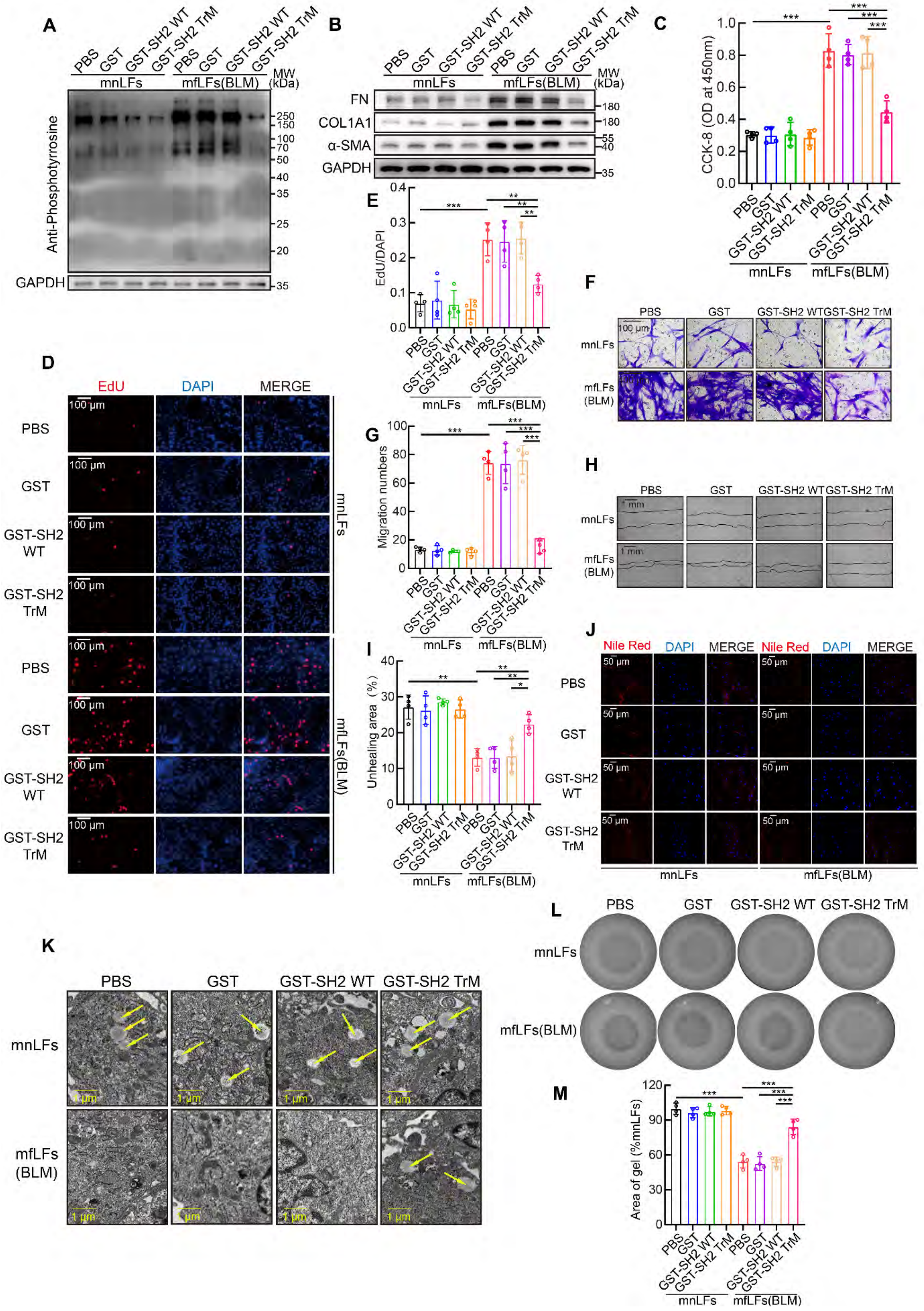
A
PBS GST GST-SH2 WT GST-SH2 TrM PBS GST GST-SH2 WT GST-SH2 TrM
mnLFs mfLFs(BLM)
MW (kDa)
Anti-Phosphotyrrosine
GAPDH
B
FN
COL1A1
α-SMA
GAPDH
C
CCK-8 (OD at 450nm)
D
EdU DAPI MERGE
100 μm
E
EdU/DAPI
F
G
Migration numbers
H
1 mm
I
Unhealing area (%)
J
Nile Red DAPI MERGE
50 μm
K
1 μm
L
M
Area of gel (%mnLFs)

Figure S5. SH2 superbinder suppressed proliferation, migration and differentiation of lung fibroblasts from mice. We isolated lung fibroblasts from lung tissues of normal or BLM-treated mice and then treated mnLFs and mfLFs with SH2 superbinder. **A-B,** Western blot of pY, FN, COL1A1 and α-SMA in mnLFs and mfLFs after treatment with GST, GST-SH2 WT or GST-SH2 TrM. **C-E,** Changes in cell proliferation between mnLFs and mfLFs after GST, GST-SH2 WT or GST-SH2 TrM incubation determined by CCK-8 and EdU assay (n = 4)**.** $^{**}P < 0.01$, $^{***}P < 0.001$. **F-I,** Comparison of migration between mnLFs and mfLFs after GST, GST-SH2 WT or GST-SH2 TrM treatment (n = 4)**.** $^{*}P < 0.05$, $^{**}P < 0.01$, $^{***}P < 0.001$. **J-K,** Differentiation measured by content of lipid droplets through TEM and Nile red staining after GST, GST-SH2 WT or GST-SH2 TrM treatment. Arrows pointed to the lipid droplets in cytoplasm. **L-M,** The contractility of mnLFs and mfLFs after GST, GST-SH2 WT or GST-SH2 TrM treatment (n = 4)**.** $^{***}P < 0.001$.

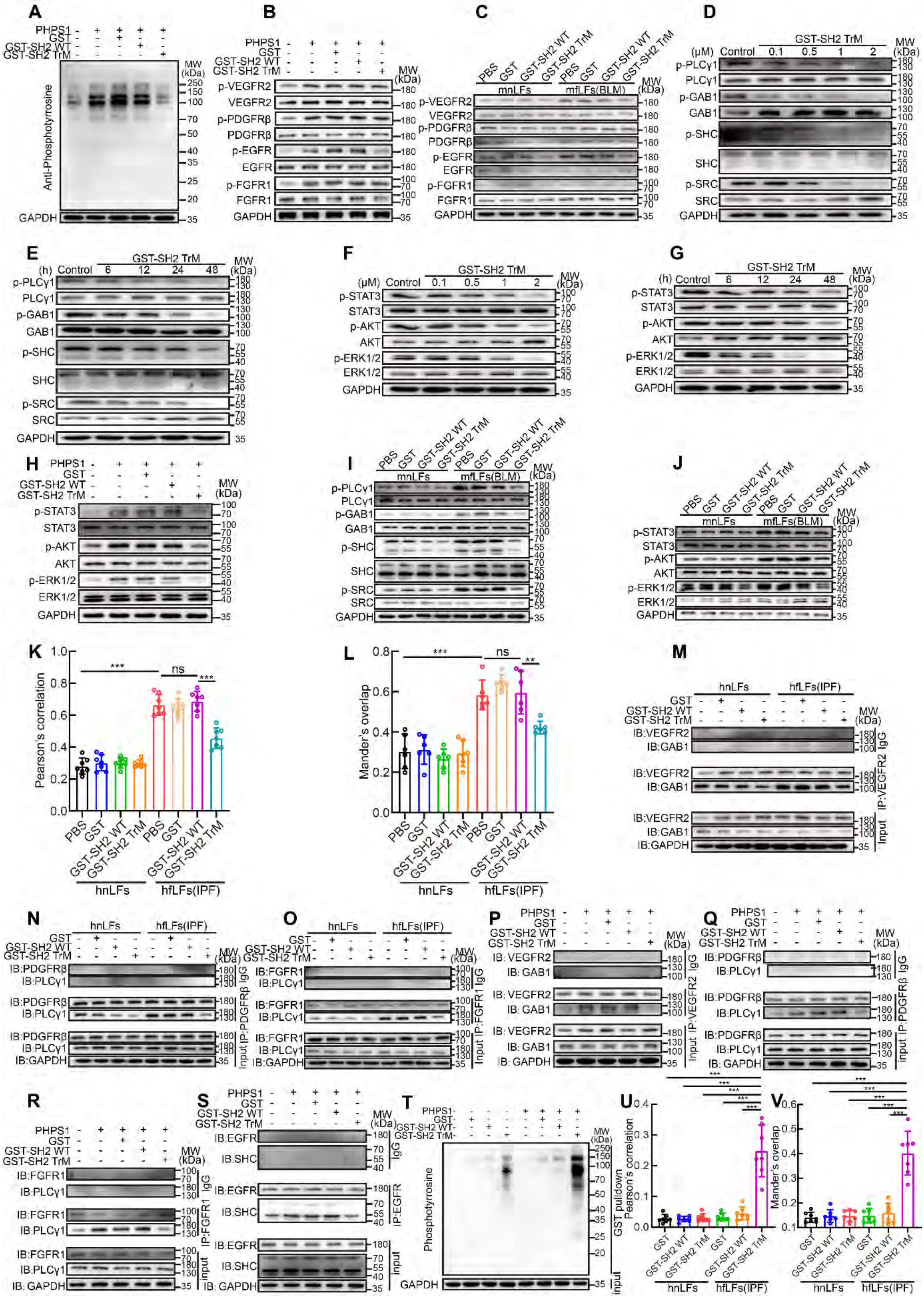

A
PHPS1
GST
GST-SH2 WT
GST-SH2 TrM
MW (kDa)
Anti-Phosphotyrrosine
GAPDH
B
p-VEGFR2
VEGFR2
p-PDGFRβ
PDGFRβ
p-EGFR
EGFR
p-FGFR1
FGFR1
GAPDH
C
PBS
GST
GST-SH2 WT
GST-SH2 TrM
mnLFs
mfLFs(BLM)
D
(μM) Control 0.1 0.5 1 2
GST-SH2 TrM
p-PLCγ1
PLCγ1
p-GAB1
GAB1
p-SHC
SHC
p-SRC
SRC
GAPDH
E
(h) Control 6 12 24 48
F
p-STAT3
STAT3
p-AKT
AKT
p-ERK1/2
ERK1/2
G
H
I
J
K
Pearson's correlation
L
Mander's overlap
hnLFs
hfLFs(IPF)
ns
M
IB:VEGFR2
IB:GAB1
IB:GAPDH
IgG
IP:VEGFR2
Input
N
IB:PDGFRβ
IB:PLCγ1
IP:PDGFRβ
O
IB:FGFR1
IP:FGFR1
P
Q
R
S
IB:EGFR
IB:SHC
IP:EGFR
T
Phosphotyrrosine
GST pulldown
input
U
Pearson's correlation
V
Mander's overlap

Figure S6. SH2 superbinder could inhibit combination of RTKs with adaptor proteins and signaling pathways downstream, which depends on pY-SH2 binding. A-B, Western blot showed the change of pY, p-VEGFR2, p-PDGFRβ, p-EGFR and p-FGFR1 in PHPS1 pretreated hnLFs after SH2 superbinder incubated. **C,** Western blot of p-VEGFR2, p-PDGFR β, p-EGFR and p-FGFR1 in mnLFs and mfLFs after treatment with GST, GST-SH2 WT or GST-SH2 TrM. **D-E,** Concentration and time dependent effects of GST-SH2 TrM on p-PLCγ1, p-GAB1, p-SHC and p-SRC protein levels in hfLFs. **F-G,** Concentration and time dependent effects of GST-SH2 TrM on p-AKT, p-ERK1/2 and p-STAT3 protein levels in hfLFs. **H,** Western blot of p-AKT, p-ERK1/2 and p-STAT3 in PHPS1 pretreated hnLFs after treatment with GST, GST-SH2 WT or GST-SH2 TrM. **I,** Western blot of p-PLCγ1, p-GAB1, p-SHC and p-SRC in mnLFs and mfLFs after treatment with GST, GST-SH2 WT or GST-SH2 TrM. **J,** Western blot of p-AKT, p-ERK1/2 and p-STAT3 in mnLFs and mfLFs after treatment with GST, GST-SH2 WT or GST-SH2 TrM. **K-L,** Co-location analysis of EGFR and SHC in SH2 superbinder treated hnLFs and hfLFs (n = 7). $^{**}P < 0.01$, $^{***}P < 0.001$. **M-O,** The immunoprecipitation of VEGFR2 and GAB1 (**M**), PDGFRβ and PLCγ1 (**N**), and FGFR1 and PLCγ1 (**O**) in hnLFs and hfLFs. **P-S,** The immunoprecipitation of VEGFR2 and GAB1 (**P**), PDGFRβ and PLCγ1 (**Q**), FGFR1 and PLCγ1(**R**), and EGFR and SHC (**S**) in PHPS1-treated hnLFs. **T,** GST pull down assay showed the binding capacity of SH2 superbinder with pY in PHPS1-treated hnLFs. **U-V,** Co-location analysis of pY and SH2 superbinder in hnLFs and hfLFs (n = 7). $^{***}P < 0.001$.

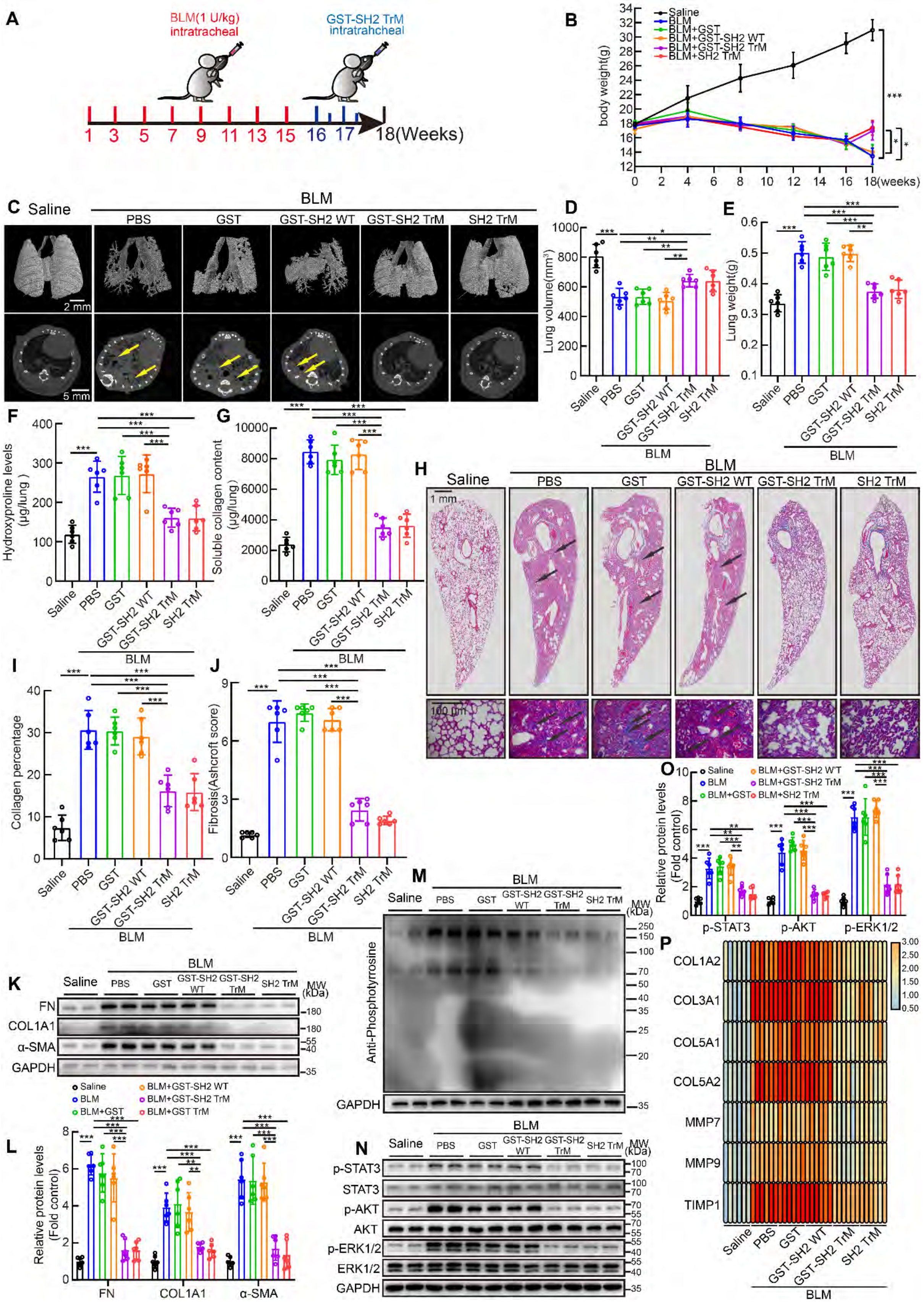
A
BLM(1 U/kg)
intratracheal
GST-SH2 TrM
intratrahcheal
1 3 5 7 9 11 13 15 16 17 18(Weeks)
B
body weight(g)
Saline
BLM
BLM+GST
BLM+GST-SH2 WT
BLM+GST-SH2 TrM
BLM+SH2 TrM
18(weeks)
C
Saline
BLM
PBS
GST
GST-SH2 WT
GST-SH2 TrM
SH2 TrM
2 mm
5 mm
D
Lung volume(mm³)
E
Lung weight(g)
F
Hydroxyproline levels
(μg/lung)
G
Soluble collagen content
(μg/lung)
H
1 mm
100 μm
I
Collagen percentage
J
Fibrosis(Ashcroft score)
K
FN
COL1A1
α-SMA
GAPDH
MW
(kDa)
L
Relative protein levels
(Fold control)
M
Anti-Phospholyrrosine
N
p-STAT3
STAT3
p-AKT
AKT
p-ERK1/2
ERK1/2
O
p-STAT3
p-AKT
p-ERK1/2
P
COL1A2
COL3A1
COL5A1
COL5A2
MMP7
MMP9
TIMP1

Figure S7. SH2 superbinder limited repetitively intratracheal BLM-induced pulmonary fibrosis in mice. After BLM (1 U/kg) treatment every two weeks for 4 months, PBS, GST, GST-SH2 WT, GST-SH2 TrM or SH2 TrM were intratracheal injected twice a week for another two weeks. All mice were sacrificed at the end of week 18. **A,** Schematic of SH2 superbinder treatment in repetitively intratracheal BLM-induced pulmonary fibrosis. **B,** Body weights measurement every 4 weeks (n = 6). $^{*}P < 0.05$, $^{***}P < 0.001$. **C,** Representative microCT reconstitution images of 3D and cross sections of lungs. Arrows showed the fibrosis foci which resulted in serious parenchymal destruction and volume reduction of lungs. **D,** Lung volume calculation by CTvox after 3D reconstitution of lungs (n = 6). $^{*}P < 0.05$, $^{**}P < 0.01$, $^{***}P < 0.001$. **E,** Lung weights shown as bar charts (n = 6). $^{**}P < 0.01$, $^{***}P < 0.001$. **F,** Lung hydroxyproline content in different groups (n = 6). $^{***}P < 0.001$. **G,** Soluble collagen content in different groups (n = 6). $^{***}P < 0.001$. **H,** Masson's trichrome staining in different groups. Images showing the panoramic and partial view of lungs. Arrows pointed to the collagen deposition. **I-J,** Collagen percentage (**I**) and fibrosis score (**J**) of lungs after PBS, GST, GST-SH2 WT, GST-SH2 TrM or SH2 TrM treatment (n = 6). $^{***}P < 0.001$. **K-O,** Western blot of FN, COL1A1 and α-SMA (**K-L**), pY (**M**), and p-STAT3, p-AKT and p-ERK1/2 (**N-O**) in lung tissues from different groups (n = 6). $^{**}P < 0.01$, $^{***}P < 0.001$. **P,** Expression changes of fibrosis associated genes after PBS, GST, GST-SH2 WT, GST-SH2 TrM or SH2 TrM treatment (n = 6).

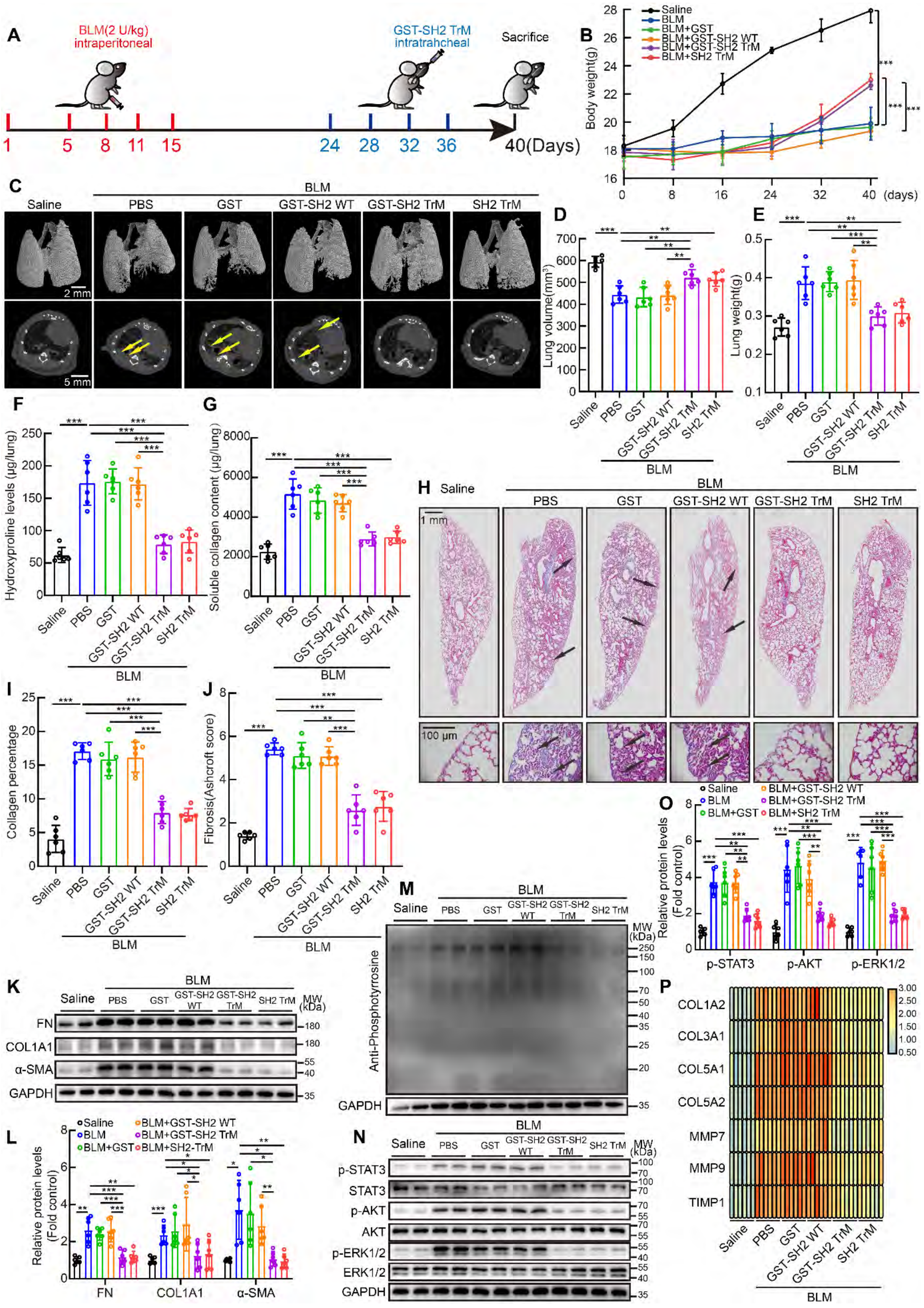
A
BLM(2 U/kg)
intraperitoneal
GST-SH2 TrM
intratracheal
Sacrifice
1 5 8 11 15 24 28 32 36 40(Days)
B
Saline
BLM
BLM+GST
BLM+GST-SH2 WT
BLM+GST-SH2 TrM
BLM+SH2 TrM
Body weight(g)
(days)
C
Saline
BLM
PBS
GST
GST-SH2 WT
GST-SH2 TrM
SH2 TrM
2 mm
5 mm
D
Lung volume(mm³)
E
Lung weight(g)
F
Hydroxyproline levels (μg/lung)
G
Soluble collagen content (μg/lung)
H
1 mm
100 μm
I
Collagen percentage
J
Fibrosis(Ashcroft score)
K
FN
COL1A1
α-SMA
GAPDH
MW (kDa)
L
Relative protein levels (Fold control)
BLM+SH2-TrM
M
Anti-Phosphotyrosine
N
p-STAT3
STAT3
p-AKT
AKT
p-ERK1/2
ERK1/2
O
BLM+SH2 TrM
p-STAT3
p-AKT
p-ERK1/2
P
COL1A2
COL3A1
COL5A1
COL5A2
MMP7
MMP9
TIMP1

Figure S8. SH2 superbinder reversed intraperitoneal BLM-induced pulmonary fibrosis in mice. After BLM (40 U/kg) treatment at day 1, 5, 8, 11 and 15, PBS, GST, GST-SH2 WT, GST-SH2 TrM or SH2 TrM were intratracheally injected at day 24, 28, 34 and 36. All the mice were sacrificed at day 40. **A,** Schematic of SH2 superbinder treatment in intraperitoneal BLM-induced pulmonary fibrosis. **B,** Body weights measurement every 8 days (n = 6). $^{***}P < 0.001$. **C,** Representative microCT reconstitution images of 3D and cross sections of lungs. Arrows showed the fibrosis foci which resulted in serious parenchymal destruction and volume reduction of lungs. **D,** Lung volume calculation by CTvox after 3D reconstitution of lungs (n = 6). $^{**}P < 0.01$, $^{***}P < 0.001$. **E,** Lung weights shown as bar charts (n = 6). $^{**}P < 0.01$, $^{***}P < 0.001$. **F,** Lung hydroxyproline content in different groups (n = 6). $^{***}P < 0.001$. **G,** Soluble collagen content in different groups (n = 6). $^{***}P < 0.001$. **H,** Masson's trichrome staining in different groups. Images showing the panoramic and partial view of lungs. Arrows pointed to the collagen deposition. **I-J,** Collagen percentage (**I**) and fibrosis score (**J**) in mice lungs of PBS, GST-SH2 WT, GST-SH2 TrM or SH2 TrM treatment (n = 6). $^{***}P < 0.001$. **K-O** Western blot of FN, COL1A1 and α-SMA (**K-L**), pY (**M**), and p-STAT3, p-AKT and p-ERK1/2 (**N-O**) in lung tissues from different groups (n = 6). $^{*}P < 0.05$, $^{**}P < 0.01$, $^{***}P < 0.001$. **P,** Expression changes of fibrosis associated genes after PBS, GST-SH2 WT, GST-SH2 TrM or SH2 TrM treatment (n = 6).

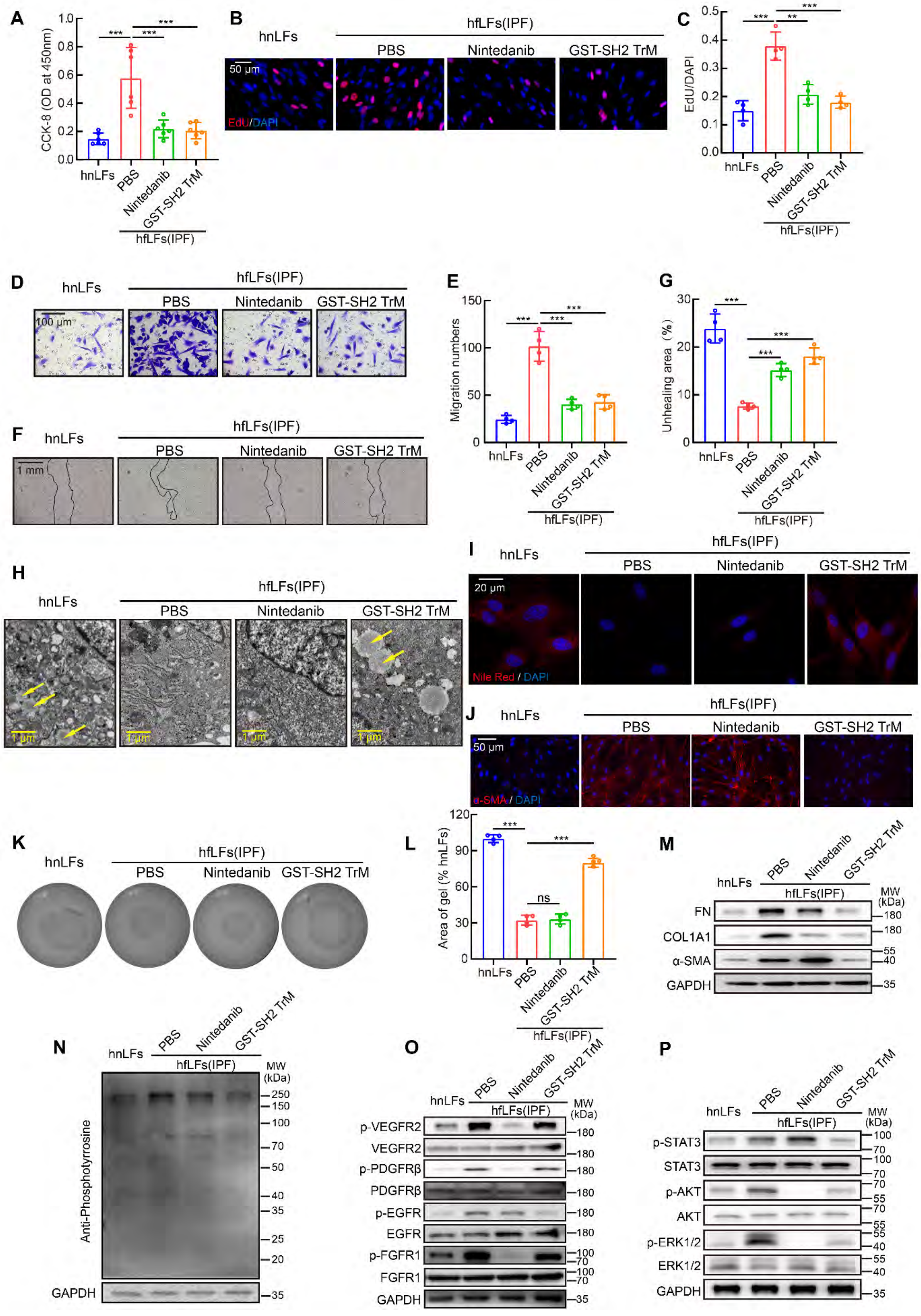
A
CCK-8 (OD at 450nm)
hnLFs
PBS
Nintedanib
GST-SH2 TrM
hfLFs(IPF)
B
50 μm
EdU/DAPI
C
EdU/DAPI
D
100 μm
E
Migration numbers
F
1 mm
G
Unhealing area (%)
H
1 μm
I
20 μm
Nile Red / DAPI
J
α-SMA / DAPI
K
L
Area of gel (% hnLFs)
ns
M
FN
COL1A1
α-SMA
GAPDH
MW (kDa)
N
Anti-Phosphotyrosine
O
p-VEGFR2
VEGFR2
p-PDGFRβ
PDGFRβ
p-EGFR
EGFR
p-FGFR1
FGFR1
P
p-STAT3
STAT3
p-AKT
AKT
p-ERK1/2
ERK1/2

Figure S9. SH2 superbinder had effective inhibition of proliferation, migration and differentiation than nintedanib. HfLFs were treated with nintedanib (1 μM) and GST-SH2 TrM (1 μM) for 24 h. **A-C,** Changes in cell proliferation between the effect of nintedanib and GST-SH2 TrM on hfLFs by CCK-8 and EdU assay (n = 6 or 4). $^{**}P < 0.01$, $^{***}P < 0.001$. **D-G,** Comparison of migration between nintedanib and GST-SH2 TrM in hfLFs (n = 4). $^{***}P < 0.001$. **H-L,** Comparison of differentiation between nintedanib and GST-SH2 TrM in hfLFs (n = 4). $^{***}P < 0.001$. **M,** Comparison of ECM production between nintedanib and GST-SH2 TrM in hfLFs. **N-P,** Comparison of pY (**N**), RTKs (**O**) and signaling pathways downstream (**P**) between nintedanib and GST-SH2 TrM in hfLFs.

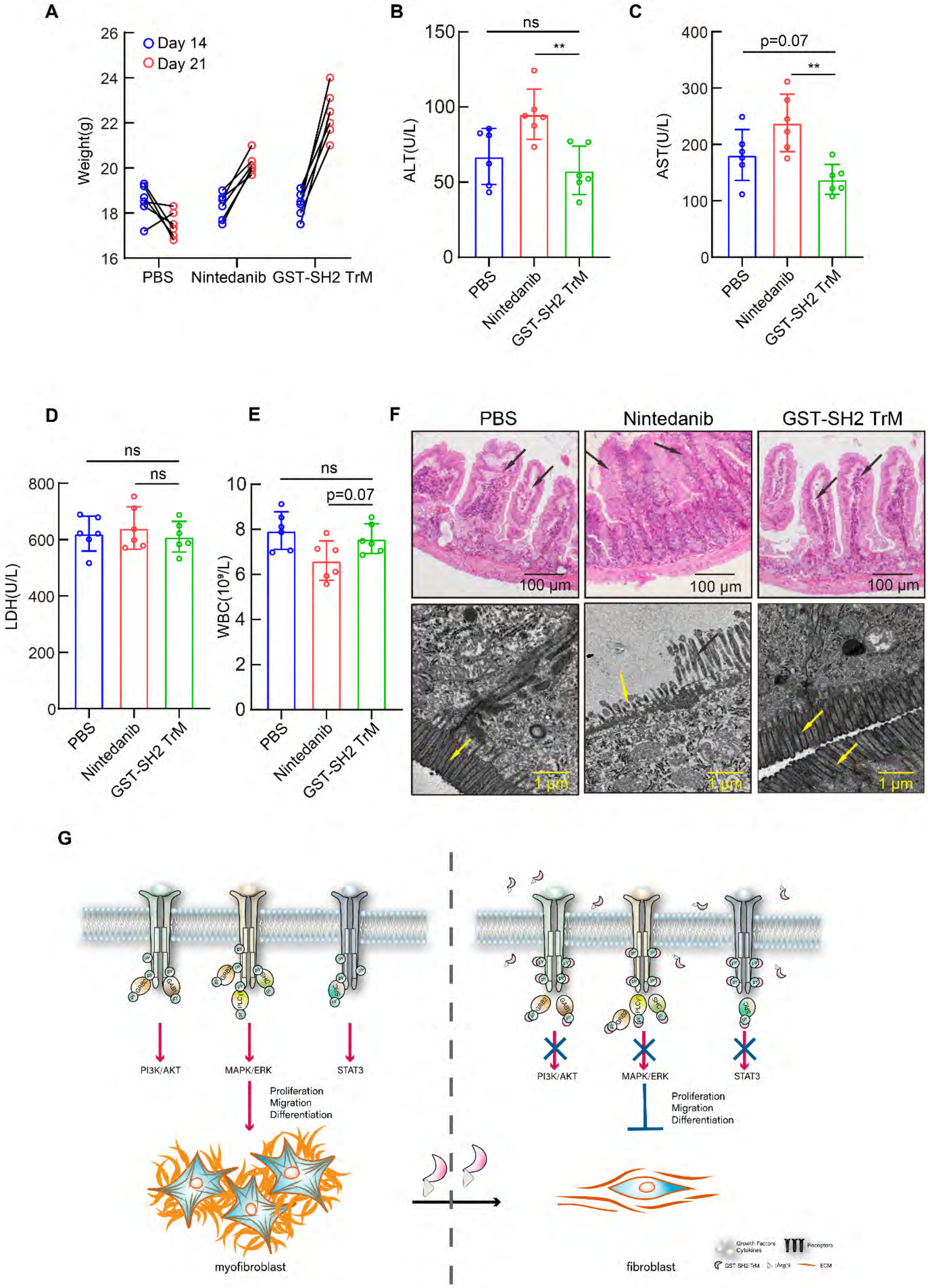
A
Day 14
Day 21
Weight(g)
PBS
Nintedanib
GST-SH2 TrM
B
ALT(U/L)
ns
**
C
AST(U/L)
p=0.07
**
D
LDH(U/L)
ns
ns
E
WBC(10⁹/L)
ns
p=0.07
F
PBS
Nintedanib
GST-SH2 TrM
100 μm
1 μm
G
PI3K/AKT
MAPK/ERK
STAT3
Proliferation
Migration
Differentiation
myofibroblast
fibroblast
Growth Factors Cytokines
Receptors
GST-SH2 TrM
ECM

Figure S10. SH2 superbinder had fewer side-effects than nintedanib *in vivo*. A, The change of body weights after PBS, nintedanib or GST-SH2 TrM intratracheal injection in BLM treated mice (n = 6). **B-D,** The determination of liver function index in serum (n = 6). $^{**}P < 0.01$. **E,** The white blood cell count after PBS, nintedanib or GST-SH2 TrM injection (n = 6). **F,** HE staining and TEM showed the difference of intestinal villi after PBS, nintedanib or GST-SH2 TrM injection. **G,** The graphical abstract on the mechanism that how SH2 superbinder inhibited the process of lung fibrosis.

Table S1. Patient demographics

	#	Age	Sex	Condition	Clinical diagnosis
Control 1	1373052	49	M	adenocarcinoma*	Lung adenocarcinoma
Control 2	1511906	64	M	adenocarcinoma*	Lung adenocarcinoma
Control 3	1494759	60	M	adenocarcinoma*	Lung adenocarcinoma
Patient 1	1361250	61	M	UIP	IPF
Patient 2	1517698	66	M	UIP	IPF
Patient 3	1562931	57	M	UIP	IPF

*normal lung tissue resected from adenocarcinoma

UIP: usual interstitial pneumonitis

IPF: idiopathic pulmonary fibrosis
